# Effects of (Poly)phenols on Circadian Clock Gene–Mediated Metabolic Homeostasis in Cultured Mammalian Cells: A Scoping Review

**DOI:** 10.1016/j.advnut.2024.100232

**Published:** 2024-04-20

**Authors:** Noha Sulaimani, Michael J Houghton, Maxine P Bonham, Gary Williamson

**Affiliations:** 1 Department of Nutrition, Dietetics and Food, Monash University, Notting Hill, Australia; 2 Victorian Heart Institute, Faculty of Medicine, Nursing and Health Sciences, Monash University, Clayton, Australia; 3 Department of Food and Nutrition, King Abdulaziz University, Jeddah, Saudi Arabia

**Keywords:** flavonoid, chronobiology, BMAL1, circadian oscillator, synchronization technique, nobiletin, cultured cells

## Abstract

Circadian clocks regulate metabolic homeostasis. Disruption to our circadian clocks, by lifestyle behaviors such as timing of eating and sleeping, has been linked to increased rates of metabolic disorders. There is now considerable evidence that selected dietary (poly)phenols, including flavonoids, phenolic acids and tannins, may modulate metabolic and circadian processes. This review evaluates the effects of (poly)phenols on circadian clock genes and linked metabolic homeostasis in vitro, and potential mechanisms of action, by critically evaluating the literature on mammalian cells. A systematic search was conducted to ensure full coverage of the literature and identified 43 relevant studies addressing the effects of (poly)phenols on cellular circadian processes. Nobiletin and tangeretin, found in citrus, (–)-epigallocatechin-3-gallate from green tea, urolithin A, a gut microbial metabolite from ellagitannins in fruit, curcumin, bavachalcone, cinnamic acid, and resveratrol at low micromolar concentrations all affect circadian molecular processes in multiple types of synchronized cells. Nobiletin emerges as a putative retinoic acid–related orphan receptor (*RORα/γ*) agonist, leading to induction of the circadian regulator brain and muscle ARNT-like 1 (*BMAL1)*, and increased period circadian regulator 2 (*PER2)* amplitude and period. These effects are clear despite substantial variations in the protocols employed, and this review suggests a methodological framework to help future study design in this emerging area of research.

**Statements of significance**

This scoping review shows that dietary (poly)phenols including nobiletin and tangeretin from citrus, EGCG from green tea, resveratrol, and curcumin are capable of regulating cellular circadian clock oscillators and metabolic homeostasis, exerting their effects partly through gene regulation. Based on the extracted data, we also provided recommendations for future work on cells, such as using physiologically relevant concentrations of (poly)phenols together with a suitable synchronization technique.

## Introduction

Lifestyle behaviors such as shortened overnight fasting, shift work, and jet lag lead to an increased risk of obesity and metabolic disorders [[1], [2], [3]], including type 2 diabetes [4] and cardiovascular diseases [5,6]. A growing body of evidence suggests that metabolic homeostasis relies on coordination with an endogenous circadian timing system, called the “biological clock” [7,8]. This system consists of the central circadian clock, also called the “master pacemaker,” as well as peripheral clocks. Through various signaling pathways, the central clock, located in the hypothalamic suprachiasmatic nucleus (SCN) of the brain [9], coordinates and synchronizes all peripheral circadian clocks found in virtually all body cells [10]. Together these clocks generate circadian rhythms, which display ∼24-h oscillations and persist even in the absence of external cues defined as zeitgebers [7,11], a German term that translates to “time givers.” Zeitgebers, such as light, eating, physical activity, and body temperature, play a role in entraining and synchronizing the circadian clocks with external time [[12], [13], [14], [15]]. Although light is the dominant zeitgeber for the central clock, perceived by the retina and directly transmitted to the SCN [16], feeding is also an important stimulus that profoundly influences peripheral clocks independent of the SCN [17,18].

**FIGURE 1:**
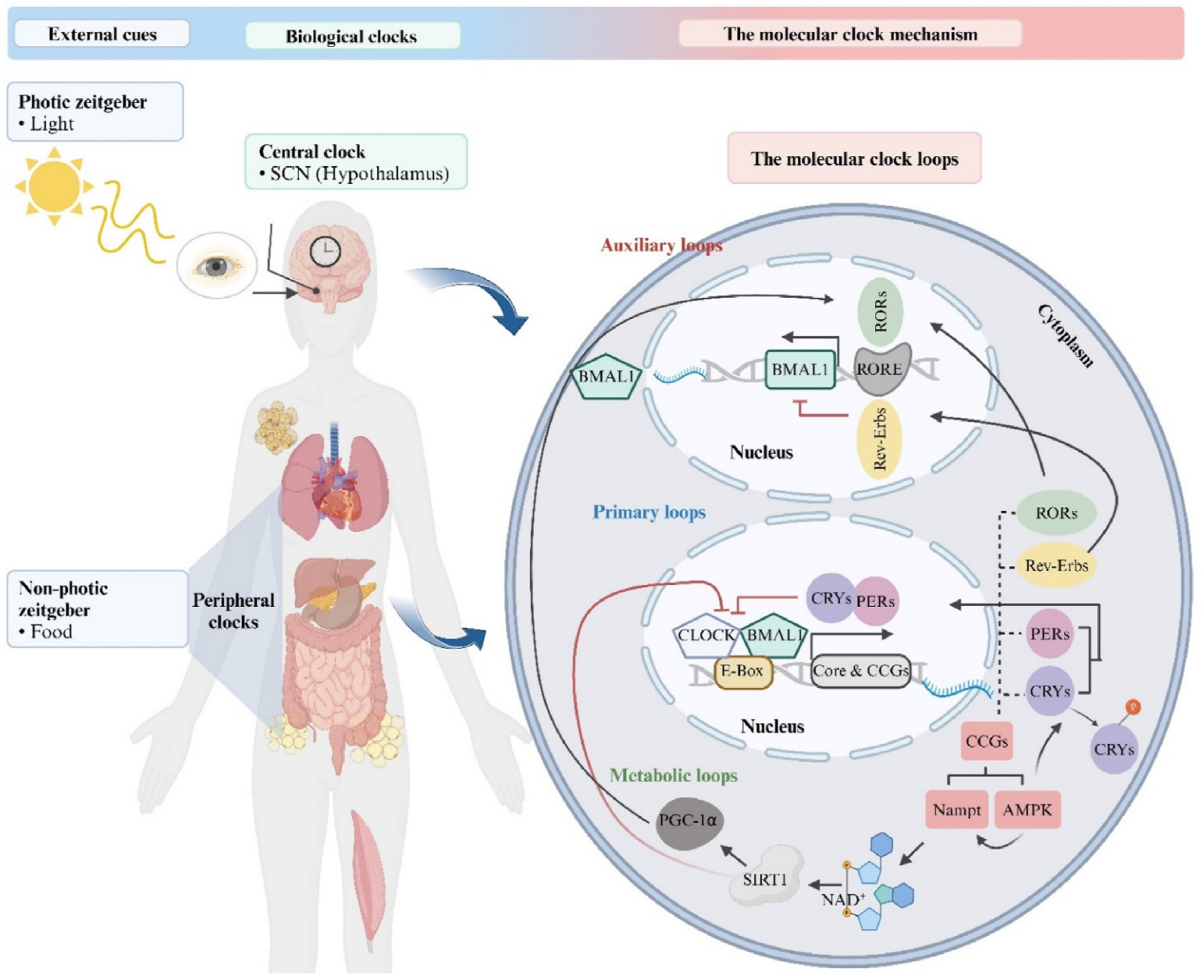
Schematic representation of the relationship between external cues and circadian clock mechanism. Information adapted from combined sources [15,20]. Abbreviations: SCN, suprachiasmatic nucleus; AMPK, adenosine monophosphate–activated protein kinase; BMAL1, brain and muscle ARNT-like 1; CLOCK, circadian locomotor output cycles kaput; CCGs, clock-controlled genes; CRYs, cryptochromes; NAD^+^, nicotinamide adenine dinucleotide; Nampt, nicotinamide phosphoribosyltransferase; PERs, periods; PGC-1α, peroxisome proliferator–activated receptor gamma coactivator 1 alfa; Rev-Erbs, reverse erythroblastosis viruses; RORs, retinoic acid–related orphan receptors; RORE, ROR enhancer elements; SIRT1, sirtuin1. Created with https://www.biorender.com/.

To generate a 24-h oscillation, the circadian rhythm is governed by an intricate network of coordinated clock factors. At the molecular level, central and peripheral circadian clocks are operated by the same regulatory mechanisms in all mammals (Figure 1) [15,19,20]. The circadian clock in individual cells is formed by autoregulatory transcriptional–translational feedback loops (TTFL), which include 2 activators, circadian locomotor output cycles kaput (*CLOCK*) and brain and muscle ARNT-like 1 (*BMAL1*), also known as aryl hydrocarbon receptor nuclear translocator-like protein 1 (*ARNTL*), and also 2 inhibitors, period (*PER1*, *PER2*, and *PER3*) and cryptochrome (*CRY1* and *CRY2*) [[21], [22], [23], [24]]. These 4 genes are the core components of the primary loop, and their intracellular localization and stability are governed by kinases and phosphatases [[25], [26], [27]]. After forming heterodimers in the cytoplasm, CLOCK/BMAL1 binds to the E-box sequences in the target gene promotors in the nucleus and serves as a positive feedback loop of PER/CRY transcriptional expression. When accumulating to a critical threshold, the PER/CRY heterodimer protein translocates to the nucleus and blocks the activity of CLOCK/BMAL1, thereby downregulating its own expression in a negative feedback loop. Moreover, the CLOCK/BMAL1 heterodimer is responsible for inducing an additional TTFL, comprising the nuclear receptors: retinoic acid–related orphan receptors (RORα/β/γ) and reverse erythroblastosis viruses (Rev-Erbα/β), also known as nuclear receptor subfamily 1 group D member 1/2 (NR1D1/2). These receptors regulate the transcription of *BMAL1* by binding to ROR enhancer elements (ROREs) in the *BMAL1* promoter [28]. Unlike RORs, Rev-Erbs function as a repressor of BMAL1 gene expression via competitive inhibition of RORs binding with ROREs. Rhythmic changes in RORE occupancy, mediated by RORs and Rev-Erbs, form an additional auxiliary loop of regulation, triggering the rhythmic oscillation of *BMAL1* [29].

In addition to the aforementioned genes, there are other genes known as clock-controlled genes (CCGs) that have clock-dependent expression and are cell-type specific, which are involved in a variety of metabolic processes. Many are transcriptionally triggered by the CLOCK/BMAL1 heterodimer. Nicotinamide phosphoribosyltransferase (*Nampt*) is one of the genes controlled by the circadian clock and is an essential enzyme responsible for nicotinamide adenine dinucleotide (NAD^+^) production needed for cellular metabolism. NAD^+^ induces sirtuin1 (SIRT1), which in turn is a regulatory component of the molecular clock by suppressing CLOCK/BMAL1 activity. Thus, Nampt indirectly affects the circadian clock via increased NAD^+^ concentrations [30,31]. In contrast, SIRT1-mediated activation of peroxisome proliferator–activated receptor gamma coactivator 1 alfa (PGC-1α), a key regulator of gluconeogenesis and glycolysis, functions as a transcriptional coactivator for *BMAL1* by acting in concert with RORα [32]. Furthermore, adenosine monophosphate–activated protein kinase (AMPK), serving as a cellular energy sensor by producing ATP and repressing ATP-consuming pathways in conditions of energy depletion, contributes to synchronization of peripheral clocks through physiological processes [8]. Alongside its role in Nampt activation [33], AMPK is essential for phosphorylating and destabilizing CRY1 to adjust the clock [27,34]. Similar to AMPK, casein kinases 1δ/ε (CK 1δ/ε) influence the stability of PERs [26,35]. This confirms the reciprocal link between circadian clocks and the regulation of metabolism via CCGs.

Circadian misalignment results from the desynchronization of circadian clocks by inappropriately timed external stimuli [7]. Modern life, with artificial light and endless social opportunities, has allowed us to be awake and active at night, contributing to the misalignment of behavioral rhythms to the endogenous clock. Another important contributing factor is extending eating occasions over the biological day (i.e., eating later at night), thereby shortening the overnight period of fasting [2]. Such behavioral disruption of the circadian rhythm presents as perturbations in glucose and lipid metabolism [7,36]. This can be attributed to the differential regulation of nutrient metabolism across the day, characterized by lower glucose [36] and lipid clearance [37] at night, owing to the activation of fewer genes involved in metabolic pathways. Similar to the impact of meal timing on gene expression, macronutrient meal composition has also been proposed to affect peripheral clocks and key metabolic factors, such as the regulation of REV-ERBα gene expression and glucagon secretion by glucose through the AMPK–Nampt–SIRT1 pathway [28]. Thus, the timing of meals, and the content of meals consumed at an inappropriate time, may adversely influence metabolic response to food [38,39], emphasizing the circadian–metabolic interaction.

Chrononutrition, defined as the study of appropriate timings of intake of particular dietary components [40], has been the subject of research examining the association between nutrition and metabolic regulation, based on an understanding of circadian rhythms and clock gene mechanisms [40,41]. Although nutrient components have been studied for their role in circadian regulation, nonnutrient components such as (poly)phenols are of interest due to their ability to improve biomarkers of chronic disease [15]. Regular consumption of (poly)phenols, bioactive components found abundantly in plant-based foods, lowers the risk of developing chronic diseases including obesity and type 2 diabetes [[42], [43], [44]]. Classes, structures, contents in foods, and metabolite concentrations in bodily fluids have been reported in numerous reviews and a searchable database [[45], [46], [47], [48], [49], [50]]. In light of supportive findings regarding the relationship between circadian disruption and metabolic disorders, a number of (poly)phenols have recently been investigated for their potential to impact circadian rhythms through modulating the expression of clock genes. Noteworthy, an emerging body of evidence indicates that (poly)phenols have a potential role in ameliorating metabolic disorders by restoring the oscillations of clock genes and CCGs [15].

Numerous in vitro models have been developed using either primary cells or cultured immortalized cells to examine the effect of bioactive molecules on clock gene expression and periodicity. Most cells, either primary or immortalized, have intact circadian clock pathways and show periodicity of clock gene expression. Some cell types such as human bone osteosarcoma U-2 OS cells exhibit robust circadian patterns, whereas, for example, human breast adenocarcinoma MCF-7 present weaker oscillations, and human breast adenocarcinoma MDA-MB-231 cells are known for their arrhythmic nature [51]. These differences have been exploited in experimental design of mechanistic studies. Due to the absence of central clock signals in vitro, cultured cells are required to be synchronized to coordinate circadian rhythms in all cells in a manner similar to that in vivo [52]. Cell synchronization refers to a technique by which all cells are brought into the same phase of expression of circadian clock genes and then sustain this synchronized expression across time [53,54]. The “gold standard” method is to synchronize cells for experiments. Without synchronization, the individual cellular rhythms within cultured cells in vitro are not coordinated, leading to a lack of overall synchronization in circadian gene expression and ultimately make it difficult to study the gene expression patterns and physiological processes regulated by the circadian clock. However, the intracellular mechanisms and transcription factors required to facilitate the effects of (poly)phenols on gene expression of clock genes are present in the cell whether or not synchronization has been carried out. Synchronization will mainly affect the quantitative and temporal aspects, but we make the assumption that synchronization will not turn off the pathways, only affect their magnitude. Therefore, we included both experiments on synchronized and unsynchronized cells in the review. Using the data from the selected papers, we could not compare the magnitude of induction due to the different scales between bioluminescence, always used with synchronization techniques, and quantitative PCR. For example, we checked data on synchronized and unsynchronized HepG2 cells and rat-1 fibroblasts, but it was not possible to quantify the differences because of variations in time points, types of (poly)phenols used, and scale of the plotted data. In vitro, several synchronization approaches have been reported, including temperature cycles, chemical reagents, mechanical stimuli, and oxidative/hypoxic stress [52]. Chemical approaches such as dexamethasone (Dex) [55], forskolin (Fsk) [56], and a high concentration of serum termed a “serum shock protocol” [57], have been used to induce the oscillatory patterns of clock genes in cultured cells. Owing to the efficacy of 50% horse serum, Dex, Fsk, and epidermal growth factor (EGF) in generating high-amplitude oscillations in rat-1 fibroblast cells compared with other reagents tested [58], these chemical synchronizers are the predominant methods. To monitor rhythmic oscillations in vitro, various methods are utilized including chronological collection, bioluminescence reporter gene constructs, and fluorescent fusion proteins. Following the observed rhythmic data, it is important to not only consider gene expression variation over time but also to analyze features of periodicity, described as amplitude, period, and phase, for a better understanding of rhythmic pattern [52]. The measurement and analysis of rhythmicity in cells involves multiple approaches and techniques. This methodology will not be reviewed here but has been described in several excellent reviews [[59], [60], [61], [62], [63], [64]].

(Poly)phenols have the potential to affect chronobiology, including biological rhythms and biological timing mechanisms [65], either directly or through effects on metabolism. A preliminary search indicated a lack of publications on the effect of polyphenols on chronobiological processes in human intervention studies. Hence, we decided to focus on in vitro studies and hope that this will provide a stimulus for future intervention studies. The aim of this article is therefore to provide a comprehensive assessment and evaluation, based on the available literature, of how (poly)phenols may regulate metabolic homeostasis via circadian rhythms through experiments on mammalian cells in vitro. This review provides insight into the potential mechanisms whereby (poly)phenols can influence the circadian rhythmicity of clock components and metabolism, and provide critically evaluated information needed for (poly)phenols to be incorporated into future human intervention studies examining their potential for improving circadian–metabolic health. Specifically, the following research questions have directed this scoping review:

• What is the role of (poly)phenols on the circadian rhythmicity of clock genes?
• What are the impacts and potential molecular mechanism(s) of (poly)phenols on modulating clock-mediated metabolic homeostasis?

## Methods

### Protocol and registration

The review protocol was performed following the guidelines of the Preferred Reporting Items for Systematic reviews and Meta-Analyses extension for Scoping Reviews (PRISMA-ScR) [66]. The preliminary protocol was reviewed and revised as necessary by the authors. It was registered prospectively with the Open Science Framework (OSF) on 7 February 2023 and is available at https://doi.org/10.17605/OSF.IO/DVGX4.

### Eligibility criteria

Relevant studies were included according to the inclusion and exclusion criteria developed based on the Population, Intervention, Comparator, and Outcome framework, along with the source of evidence and language (Table 1). Papers addressing the effect of (poly)phenols, (poly)phenol-rich extracts or mixtures on circadian clock genes and involving primary and immortalized mammalian cell lines in vitro were included. The sources of evidence taken in this review were limited to primary research articles. Due to the broad nature of the concept of circadian clock processes, the inclusion criteria were refined to encompass exclusively circadian clock genes. In addition, solely English-language papers were considered.

**TABLE 1:**
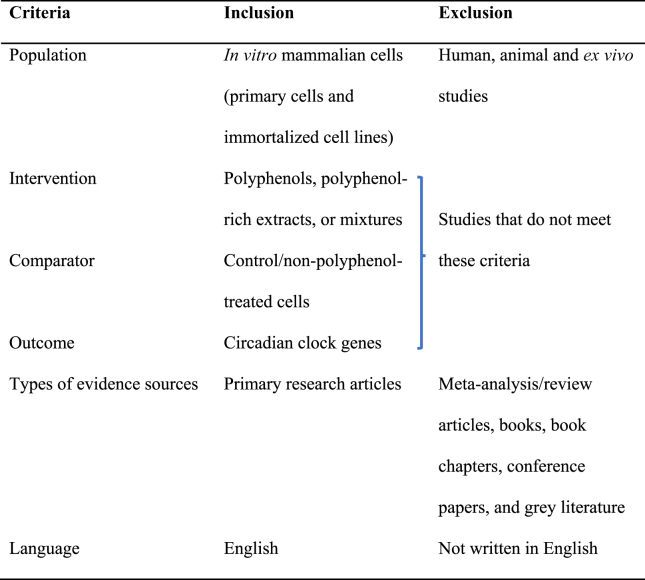
Inclusion and exclusion criteria

### Information sources and search strategy

A date-unrestricted search of the Ovid Medline, Web of Science, and Scopus databases was conducted on 12 December 2022, with updates on 11 January 2023 and 13 March 2024. The MeSH headings and keywords were first determined in Medline and subsequently applied to the other databases, with necessary modifications to meet the specific needs of each database (see full electronic search strategy for databases in Supplemental Table 1). Boolean and Proximity operators were employed to combine chosen keywords and limit the number of publications. To test our search, a gold set of relevant articles, representing the review question, was previously identified to ensure their retrieval. An initial search was performed using 3 core concepts, namely, (poly)phenols, circadian clock genes and mammalian cell lines. Due to the limited number of pertinent papers, the inclusion of the third concept, mammalian cell lines, as a search criterion was excluded in an effort to broaden the search outcomes and this allowed specific selection of papers on mammalian cell lines during the screening stage. This scoping review methodology was employed to ensure that all relevant papers in the area were included.

### Selection of sources of evidence

Covidence software (https://www.covidence.org) was used for reference management of the search, duplicate removal, title and abstract screening, full-text reviewing, and data extraction. Reference review at each stage was conducted by 2 independent reviewers (N.S. and either M.H. or M.B. or G.W.), and disagreements on study selection were resolved by a third reviewer (either M.H. or G.W.).

### Data charting process

Data relevant to the review question were extracted from all included studies and recorded in a structured table using Microsoft Excel (Version 2019) and categorized by author(s), year of publication, experimental model, (poly)phenol treatment, synchronization method, condition, type/duration, comparator, outcome measures, genetically modified circadian genes, and key findings related to circadian effects and metabolic effects. It was developed by N.S. and checked for accuracy by M.H. and G.W.

### Synthesis of results

The studies were grouped by various parameters including type of (poly)phenols and type of cell. Extracted data from included studies were tabulated to facilitate interpretation. We relied on the statistical analyses used in the original papers.

## Results and Discussion

### Selection of sources of evidence

**FIGURE 2:**
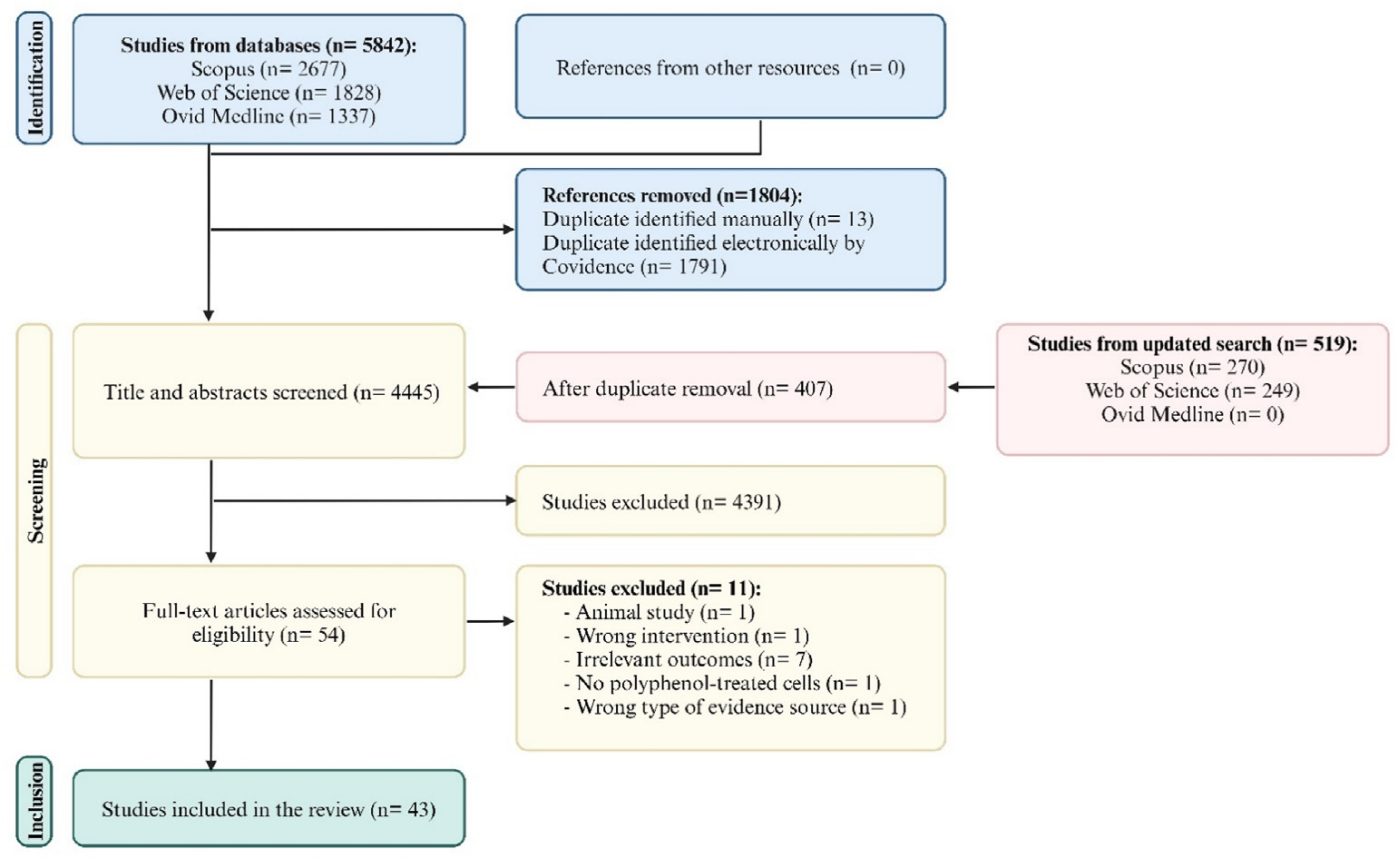
PRISMA flow diagram of the selected studies. Created with https://www.biorender.com/.

The PRISMA-ScR flow diagram of the selected studies is depicted in Figure 2. A total of 6361 articles were identified by the systematic search strategy. Following duplicate removal, 4445 studies were included in title and abstract screening, of which 54 were reviewed for full-text eligibility. Ultimately, 43 papers were eligible for inclusion.

### Characteristics of sources of evidence

The studies included in this review were published from 2008 to 2024, with the majority published after 2016, reflecting the growing scientific interest in (poly)phenols and circadian biology. Table 2 [[67], [68], [69], [70], [71], [72], [73], [74], [75], [76], [77], [78], [79], [80], [81], [82], [83], [84], [85], [86], [87], [88], [89], [90], [91], [92], [93], [94], [95], [96], [97], [98], [99], [100], [101], [102], [103], [104], [105], [106], [107], [108], [109]] summarizes the main characteristics and outcomes of the included studies.

**TABLE 2:** Summary of study characteristics

Author and year	Polyphenols	Concentration (μM)	Experimental cell model	Synchronization method	Condition (type/duration) and comparator	Genetically modified circadian gene	Key outcomes	
							Circadian effects^1^	Metabolic effects^2^
Kapar et al. [103], 2024	Resveratrol (Res)	100	Young and old human adipose progenitor cells (APCs)^3^	FBS (30%, 2 h)	Treatment with Res/12 h, collection every 6 h for 48 h between 12 and 60 h time points. Control (ethanol)	N/A	↑ SIRT1 mRNA in both cells.↑ NR1D2, CRY2, and RORα; Δ CLOCK, CRY1, and NR1D1 mRNAs in young cells at different time points. ↓CLOCK; ↑ BMAL1; Δ PER1, CRY1/2, RORα, and NR1D1/2 mRNAs in old cells at different time points. ↑ NR1D1/2 amplitude, DBP period and delay NR1D1 in young cells. ↑ NR1D1 amplitude and delayed phase of PER1 and NR1D2 in old cells. No Δ BMAL1, PER1, NR1D1/2 and DBP rhythmicity in both cells. PER2, CRY2 and RORα lost rhythmicity in both cells. Δ CLOCK and CRY1 rhythmicity in both cells	
Jiang et al. [104], 2023	Resveratrol (Res)	50	Human L-02 cells^4^	None	Combined treatment with 50 μg/mL PM_2.5_ and Res /24 h, collection every 8 h for 24 h between 24−48 h time points. Control (filtered air) and PM_2.5_.	N/A	Restorative effect: BMAL1, CLOCK, and SIRT1 proteins at various times	
Mawatari et al. [85], 2023	Sudachitin	1–10	Human U-2 OS	Dex (200 nM, 1 h)	Treatment with sudachitin, bioluminescence recording/5–7 d (using 1–10 μM), collection every 4 h for 28 h between 6 and 34 h time points for mRNA and protein analysis (using 10 μM).Control (0.1% DMSO)	BMAL1-Luc	Rhythmic oscillation of BMAL1-Luc, dose dependent. Robust ↑ amplitude (1.5- to 3-fold), dose dependent. ↑ Periods of BMAL1 at (10 μM).↑ BMAL1, PER2, and CRY1 mRNAs and proteins. ↓Rev-Erbα mRNA and protein	
			Mouse fibroblast cells			PER2::LucSV	Moderate effects on PER2 oscillations. ↑ period of PER2 only at (10 μM).Modest ↑ amplitude of PER2 (at 3 and 10 μM)	
Kirshenbaum et al. [97], 2023	Nobiletin (NOB)	25	Cardiomyocyte cells isolated from young Sprague–Dawley rats	None	Pretreatment with NOB for 6 h and then hypoxia condition. Control under normoxia and hypoxia conditions.	N/A	Restorative effect: ↑ RORα protein	
Kim et al. [83], 2023	Nobiletin (NOB)	10 or 20	Mouse embryo preadipocyte (3T3–L1)	Dex (200 nM, 1 h)	Treatment with NOB, bioluminescence/4 d (using 10 μM) using, collection every 4 h for 24 h between 0 and 20 h time points for mRNA analysis. Control (0.1% DMSO)	BMAL1:Luc	Robust rhythmic oscillation of BMAL1-Luc. ↑ BMAL1 mRNAs.↑ PER2, CRY1, DBP, and DEC1/2 mRNAs, dose dependent.Δ NR1D1 mRNA	
Hang et al. [92], 2023	7,8-Dihydroxy-flavone (7,8-DHF)	10	Neonatal primary mouse cardiac fibroblasts (CFs)	None	Treatment with TGF-β1 and 7,8-DHF/48 h.Control and TGF-β1.	N/A	↓ BMAL1, PER2, and CRY2, no Δ CLOCK proteins.	
Zou et al. [94], 2022	Resveratrol (Res)	50	Mouse LS8	None	Pretreatment Res/6 h and then 1 mM NaF/24 h. Control and NaF.	N/A	↑ CLOCK, BMAL1, and PER2 proteins.	
Ye et al. [72], 2022	Resveratrol (Res)	50	Human HK-2	None	Pretreatment Res/ 24 h then exposure to H/R conditions. siCTRL+DMSO+H/R, siCTRL+RES+H/R, and siBMAL1+DMSO+H/R	siRNA-BMAL1		↓ %Apoptosis and ↑ MMP via ↓ caspase-3 and cytoplasmic cyt c proteins. ↑ mtDNA quantity, SIRT1 and PGC-1α mRNAs and proteins, BMAL1-dependent.
Huang et al. [80], 2022	Icariin	0.1	Human BMSCs	None	Treatment with icariin. Control (DMSO), transduced BMAL1, BMAL1^-/-^ and icariin+BMAL 1^-/-^.	BMAL1 knockdown (BMAL1^-/-^) and BMAL1 transduction (overexpression)	↑ BMAL1 mRNA and protein.	↑ mRNAs and proteins involved in osteogenic differentiation, BMAL1-dependent. ↑ Osteogenic differentiation via BMAL1-BMP2 signaling. ↑ Osteogenic differentiation-associated mRNAs and proteins with BMAL1 overexpression.
Haraguchi et al. [105], 2022	Urolithin A (ULT)	10–100	Mouse embryonic fibroblasts (MEFs)	Dex (100 nM)	Chronic ULT or EA and bioluminescence recording/6 d. Acute ULT or EA at different time points/30 min and bioluminescence recording/4–5 d. Control (0.1% DMSO).	PER2::Luc and BMAL1-Eluc knock-in	Chronic: for PER2::Luc and BMAL1-Eluc, ↑ period and delayed first peak, dose dependent. ↑ Amplitude with ↓ concentrations (10 μM with BMAL1-Eluc; 10 and 20 μM with PER2::Luc). Acute: delay the phase of PER2, dose and time dependent.	
	Ellagic acid (EA)	10–100					Chronic: ↑ period and ↓ amplitude of PER2 with ↑ concentration (100 μM).Acute: delay the phase of PER2, dose and time dependent	
Huh et al. [86], 2022	*Quercus gilva* extracts: 95% ethanol or hot water extract of leaf, branch, kernel, and pericarp	N/A	Human HaCaT	Dex (100 nM, 2 h)	Treatment with ethanol gilva extract or hot water gilva extract. Control	Mouse BMAL1 promoter-luciferase	Ethanol extracts: ↑ amplitude of BMAL1, dose dependent, especially the branch extract	
Manocchio et al. [99], 2022	Grape seed proanthocyanidins extract (GSPE)	N/A	Human HepG2	2-h Serum shock (50% horse serum)	Treatment with GSPE, collection at 0, 3, 12, 15, 21, or 24 h. Control (ethanol)	N/A	↓ BMAL1 mRNA at ZT12	
Chen et al. [84], 2022	Tangeretin (Tan)	0.1–5	Mouse BV2	2-h Serum shock (50% FBS)	Pretreatment Tan/24 h and then LPS/8 h, collection at 10, 18, or 26 h. Control and LPS	N/A	↑ BMAL1 mRNA	
			Human HEK 293T	None	Pretreatment Tan/24 h and then LPS/8 h. Control and LPS	Co-transfection with Gal4-RORα-LBD, Gal4-RORβ-LBD or Gal4-RORγ-LBD plasmid	↓ Only RORα/γ activity, dose dependent	
			Human U-2 OS	None		BMAL1-Luc reporter in RORα/γ overexpressed U-2 OS	↑ BMAL1-Luc activity, dose dependent	
			Mouse NIH3T3	None		BMAL1-Luc reporter in RORα/γ overexpressed NIH3T3 cells	↑ BMAL1-Luc activity and BMAL1 and PER2 mRNAs, dose dependent	
Li et al. [67], 2022	Capsaicin (CAP)	50	Human HepG2	None	Combined treatment 0.4 mM OA and CAP/24 h, collection every 6 h between 18 and 42 h time points. Control and OA.	siRNA-BMAL1	Restorative effect: BMAL1, CLOCK, PER1/2, CRY1/2, and Rev-Erbα mRNAs and proteins.	↓ ROS production, BMAL1-dependent. ↓ MMP loss, BMAL1-dependent. ↓ lipid metabolism disorder by ↓ FAS, ACC, mTOR, and PGC-1α, whereas ↑ AKT and AMPK, BMAL1-dependent.
Chatam et al. [101], 2022	Resveratrol (Res)	50	Mouse AML12	Dex (1 mM, a 1-h pulse)	Treatment with Res/6 h, collection every 6 h for 24 h.Control.	N/A	↑ BMAL1 mRNA. Advance the phase of BMAL1. ↓ Amplitude of BMAL1.	
Mei et al. [78], 2021	(– )-Epigallocatechin-3-gallate (EGCG)	20	Rat nucleus pulposus cells (NPCs)	2-h Serum shock	Pretreatment EGCG/12 h for mRNA analysis or 4 h for protein analysis and then 300 μM H_2_O_2_/12 h or 24 h for protein analysis, collection between the 24 and 48 h time points at 6 h intervals. Control and H_2_O_2_	siRNA-BMAL1	Restorative effect: BMAL1, CLOCK, PER1/2, and CRY1/2 mRNAs. BMAL1 proteins.	↓ Cleaved caspase3 and Bax/Bcl-2 ratio, BMAL1-dependent.
Gabriel et al. [98], 2021	Resveratrol (Res)	10	Primary human myotubes^5^	2-h Serum shock (50% FBS)	Treatment with Res. Control.	siRNA-OPA1	↓ NPAS2 with OPA1 depletion.	
Wang et al. [87], 2021	7 Citrus fruit extracts^6^	N/A	Mouse BV-2	2-h Serum shock (50% horse serum)	Pretreatment citrus flavonoid extracts or NOB/12 h and then 0.1 μg/mL LPS/12 h. Control (0.05% DMSO) and LPS	Per2 luciferase reporter system knock-in (PER2::Luc)	Almost all flavonoid extracts ↑ PER2 mRNA	
	Nobiletin (NOB)	10					↑ CLOCK, BMAL1, PER1/2/3, CRY1/2, Rev-Erbα/β, RORα, DBP, and NPAS2 mRNAs	
Okada and Okada [95], 2020	Resveratrol (Res)	100	Human lung fibroblast cells (TIG-1-20, young cells; and TIG-1-60, old cells)	None	Treatment with Res/4 h. Control (0.1% DMSO).		↑ BMAL1 mRNAs in both types of fibroblast cells. ↑ NR1D1 and SIRT1, ↓ PER1 and SIRT6 mRNAs and ↑ BMAL1 protein in young cells. ↑ PER1 and SIRT6, ↓ SIRT1 mRNAs in old cells.	
	Quercetin (Que)	2 or 20			Treatment with Que/4 h. Control (0.1% DMSO)		↓ Dose ↑ BMAL1 mRNA in both types of cells.↑ Sirt1, Sirt6, and NR1D1 mRNAs, inverse dose–response in young cells. Both doses ↑ BMAL1 protein in young cells.↓ Doses ↑ PER1 protein in young cells. Both doses ↓ PER1 mRNA in young cells, ↑ PER1 in old cells	
	Caffeic acid	2.5 or 25			Treatment with caffeic acid/4 h. Control (0.1% DMSO)		↓ Dose ↑ PER1 mRNA in both types of cells. ↓ BMAL1 and SIRT1 l mRNAs in an inverse dose–response in young cells. ↑ BMAL1 mRNA concentrations in a dose–response manner in old cells. ↑ dose ↑ SIRT6 mRNA in both types of cells. ↑ BMAL1 protein in young cells. ↓ doses ↑ PER1 protein in young cells	
Jiang et al. [93], 2020	(– )-Epigallo-catechin-3-gallate (EGCG)	10–40	Human A549 and H1299	None	Treatment with EGCG/48 h.Control.	siRNA-CLOCK	↓ CLOCK mRNA and protein, dose-dependent	
Don et al. [81], 2020	Nobiletin (NOB)	5 or 50	Human U-2 OS (robust circadian rhythms)	Dex (100 nM, 2 h)	Treatment with NOB/6 d. Control (0.2% DMSO)	BMAL1-Luc and PER2::Luc reporter knock-in	Rhythmic oscillations of BMAL1 and PER2. ↑ Periods of BMAL1 and PER2.↓ Dose Δ the damping rate of PER2 oscillations	
			Human MCF7 (weak circadian rhythms)	18-h Starvation conditions (DMEM with 1% L-glutamine)			Rhythmic oscillations of BMAL1 and PER2.↓ Amplitude of BMAL1 and PER2	
			Human MDA-MB-231 (arrhythmic)	18-h Starvation conditions followed by 2-h serum shock (1:1 FBS and DMEM with 1% L-glutamine).			↑ Dose improves the rhythmicity of BMAL1 and PER2.	
Petrenko et al. [75], 2020	Nobiletin (NOB)	20	Human pancreatic α- and β-cells isolated from nondiabetic (ND) and type 2 diabetic (T2D) donors	Fsk (10 μM, a 1-h pulse)	Treatment with NOB. ND, T2D, and siCLOCK	BMAL1-Luc reporter knock-in and siRNA-CLOCK	↑ Amplitude of BMAL1 in T2D islets	↑ Insulin secretion in human T2D islets triggered by glucose, CLOCK-independent
Hirai et al. [96], 2019	Baicalein	10	Mouse (C2C12)	None	Treatment with baicalein/6 h. Control (0.1% DMSO).	siRNA-RORα	↑ RORα.↓ RORα and BMAL1 in RORα-silenced cells.	Regulate FGF21 expression, RORα dependent.
Yamamoto et al. [107], 2019	Piceatannol or passion fruit seeds extract (PFSE)	100	MEFs	Dex (200 nM, 2 h)	Transient piceatannol or PFSE/30 min at CT4 or CT20.Control (PBS).	PER2::Luc knock-in	↑ Amplitude and advance the peak phase at CT4. ↑ PER2 regardless of treatment time.	
Lu et al. [68], 2019	Capsaicin (CAP)	50	Human HepG2	None	Cotreatment 20 mM glucosamine and CAP/18 h, collection every 6 h between 18 and 42 h time points. Control and glucosamine.	siRNA-BMAL1	Restorative effect: CLOCK, BMAL1, PER1/2 and CRY1/2 mRNAs and proteins.	↓ Oxidative stress levels via ↓ ROS production and ↑ mitochondrial function, BMAL1-dependent. ↑ Glucose metabolism by ↑ glucose uptake, BMAL1-dependent.
Li et al. [76], 2019	Resveratrol (Res)	100	Human HepG2	2-h Serum shock (50% horse serum)	Pretreatment Res/6 h and then 100 μM FFA (OA: PA = 2:1)/24 h, collection at 6 h intervals between 32 and 56 h time points. Control and FFA.	siRNA-BMAL1	↑ CLOCK, BMAL1, PER1/2, CRY1, and Rev-Erbα mRNAs. ↑ CLOCK, BMAL1 and SIRT1 proteins.	↑ Enzymes involved in energy metabolism (p-AMPK, p-GSK-3β), BMAL1-dependent. ↑ Lipid metabolism via ↓ FAS, SREBP-1c, and PPARγ and ↑ p-ACC, BMAL1-dependent.
Tan et al. [69], 2019	Resveratrol (Res)	50	Human HepG2	None	Pretreatment Res/4 h and then 5 mM ACR/24 h. Control, ACR, and Res.	siRNA-BMAL1; siRNA-CRY1		↓ Cellular oxidative stress via ↑ Nrf2 and NQO-1 proteins, BMAL1-dependent.↑ mitochondrial function via ↑ mitochondrial respiration complex proteins and mRNAs involved in OXPHOS (Ndufs1 and Cox6c), BMAL1-dependent.↑ inflammatory responses via ↓ p-NF-κB, p-I-κB and inflammatory mediator mRNAs (TNF-α, iNOS, and IL-6), CRY1-dependent.
			Primary mouse hepatocytes	2-h Serum shock (50% horse serum)	Pretreatment Res/4 h and then 5 mM ACR/24 h, collection at 4 h intervals between 30 and 50 h time points. Control, ACR, and Res	N/A	↑ BMAL1, CLOCK, SIRT1, and CRY1 proteins. ↑ SIRT1 mRNA	
Qi et al. [73], 2018	Nobiletin (NOB)	200	Human HepG2	None	Pretreatment NOB/4 h and then PA/18 h.Control and PA.	N/A	↑ CLOCK and BMAL1 proteins.	
			Primary mouse hepatocytes	2-h Serum shock (50% horse serum)	Pretreatment NOB/4 h and then PA/18 h, collection at 4 h intervals between 24 and 44 h time points. Control and PA	siRNA-BMAL1	Restorative effect: CLOCK, BMAL1, PER2, CRY2, and Rev-Erbα mRNAs	↓ Glucose metabolism disorders via ↑ p-IRS-1, p-AKT, membrane-GLUT2 translocation, p-GSK-3β, and p-CREB), BMAL1-dependent. ↓ Lipid metabolism disorders via ↑ p-AMPK and p-ACC, ↓ FAS and SREBP-1c, BMAL1-dependent
Guo et al. [77], 2018	Chicoric acid (CA)	200	Human HepG2	2-h Serum shock (50% horse serum)	Cotreatment 100 μM free fatty acids (OA 2:1 PA) and CA/24 h, collection at the interval of 8 h between 28 and 52 h time points. Control (DMSO) and FFA.	siRNA-BMAL1; siRNA-CLOCK	Restorative effect: BMAL1, CLOCK, PER1/2, CRY1/2 and Rev-Erbα mRNAs.	↑ SIRT1 and PGC-1α proteins, BMAL1- and CLOCK-dependent. ↓ Lipid metabolism disorders via ↓ FAS, ↑ p-AKT and p-GSK3β proteins and ↑ PPARα mRNA, BMAL1-dependent. ↓ Intracellular lipid accumulation, BMAL1 dependent.
Qi, et al. [70], 2018	Tea polyphenols	N/A	Human HepG2	2-h Serum shock (50% horse serum)	Pretreatment TP/12 h and then 200 μM H_2_O_2_/12 h, collection at 6 h intervals between 24 and 48 h time points. Control and H_2_O_2_.	siRNA-BMAL1	Restorative effect: CLOCK, BMAL1, PER1/2, and CRY1/2 mRNAs. CLOCK, BMAL1, and SIRT1 proteins.	↓ Mitochondrial dysfunction via ↑ mitochondrial complex and stress-responsive proteins, almost all mRNAs of OXPHOS and specific markers related to mitochondrial dynamics, BMAL1 dependent. ↓ Cellular redox imbalance via ↑ the Nrf2/HO-1 antioxidant defense pathway, BMAL1 dependent.
			Primary mouse hepatocytes	None	Pretreatment TP/12 h and then 500 μM H_2_O_2_/12 h, collection at 6 h intervals between 24 and 48 h time points. Control and H_2_O_2_			↓ Mitochondrial dysfunction via ↑ mitochondrial complex and stress-responsive proteins, BMAL1 dependent
Qi et al. [71], 2017	Tea polyphenols	N/A	Human SH-SY5Y	2-h Serum shock (50% horse serum)	Pretreatment TP/12 h and then 100 μM H_2_O_2_/12 h, collection at 6 h intervals between 24 and 48 h time points. Control and H_2_O_2_	siRNA-BMAL1	Restorative effect: CLOCK, BMAL1, PER1/2, and CRY1/2 mRNAs. CLOCK, BMAL1, and SIRT1 proteins	↓ ROS generation, BMAL1 dependent via ↑ Nrf2/HO-1 antioxidant defense pathway. ↑ Mitochondrial functions via ↑ mitochondrial complex and stress-responsive proteins, BMAL1-dependent. ↓ Neuronal cell apoptosis via ↑ p-AKT, p-CREB, and BDNF, BMAL1 dependent
Shinozaki et al. [106], 2017	Flavone, 5-hydroxy-flavone, 7-hydroxy-flavone, chrysin, baicalein, apigenin, luteolin; galangin, kaempferol, quercetin, myricetin; daidzein, genistein, epicatechin, epigallo-catechin, epigallo-catechin-3-gallate;nobiletin, tangeretin	10 (Continuous treatment); or 0–200 (dose–response)	MEFs	Dex (100 nM, 2 h)	Continuous flavonoid and bioluminescence monitoring/4 d. Control (0.25% DMSO)	PER2::Luc knock-in	7-Hydroxyflavone delays first peak at 10 μM. Each flavonoid has a different dose-dependent effect on the period and amplitude of PER2. NOB and Tan ↑ the amplitude and period of PER2	
					Transient flavonoid at a specific time point (CT14) between the first and second peak/30 min. Control (0.25% DMSO).		Flavones, flavonols, isoflavones, NOB and Tan delays the phase of PER2, dose-dependent. Catechins have no Δ in the rhythm and phase of PER2.	
Mi et al. [74], 2017	(–)-Epigallocatechin-3-gallate (EGCG)	50	Human HepG2	2-h Serum shock (50% horse serum)	Cotreatment 20 mM glucosamine and EGCG/18 h, collection at 6 h intervals between 18 and 42 h time points. Control and glucosamine.	siRNA-BMAL1	Restorative effect: CLOCK, BMAL1, PER1/2, and CRY1/2 mRNAs.CLOCK, BMAL1, and SIRT1 proteins.	↓ Glucose metabolism disorder by ↑ p-IRS-1, p-AKT, GLUT2, p-AMPK, p-GSK3β, glucose uptake, and glycogen synthesis, BMAL1 dependent.
		10	Primary mouse hepatocytes					
Oishi, K. et al. [100], 2017	Cinnamic acid	0.01–10	Primary mouse neuronal cells	Fsk (10 μM)	Treatment cinnamic acid at time 0 h. Control (DMSO)	PER2::LUC knock-in mice	↓ Period of PER2, dose-dependent. ↓ Dose sustains an amplitude (> 5 d). No Δ BMAL1, PER1/2 and Rev-Erbα mRNA	
Du Pré et al. [108], 2017	Resveratrol (Res)	0.25 or 2.5	Neonatal rat cardiomyocytes (nrCMs)	2-h Serum shock (50% horse serum)	Treatment with Res.Control.	PER2-dLuc lentiviruses transduction	↓ Amplitude and ↑ period of PER2, dose dependent.	
				Fsk (10 μM, 30 min)				
				Dex (100 nM, 30 min)				
Sarma et al. [88], 2016	Curcumin (CUR)	5 or 10	Rat C6	Fsk (20 μM, 2 h)	Treatment with CUR/12 h. Control (DMSO)	mPER2:: mPER2::Luc	↓ Dose causes a rhythmic pattern of mPER2 with an average period of 24.48 h for PER2	
He et al. [82], 2016	Nobiletin (NOB)	0.3–10; 1.5–50 (Depends on experiment)	Adult mouse ear fibroblasts, MEFs, murine Hepa1-6, Human U-2 OS, and Human HEK-293T	Fsk (5 μM, 1 h) or Dex (100 nM)	Treatment with various polyphenols, collection for mRNA, and proteins at 4 h intervals between 0 and 32 h time points. Control (DMSO)	PER2::Luc and PER2::LucSV reporter fibroblast cells;PER2::Luc Clock^Δ19/Δ19^ (mutant form); PER2::Luc Clock^Δ19/+^ reporter cells; mutant RORE reporter Hepa1-6 cells; siRNA-RORα/γ Hepa1-6 and U-2 OS cells	↑ Amplitude and period of PER2::LucSV, dose- and CLOCK-dependent. ↑ BMAL1 promoter–driven Luciferase expression in a RORα/γ-dependent. Binds to RORα/γ. Δ transcripts of CLOCK, BMAL1, NPAS2, PER2, CRY1/2, RORα/γ, and Rev-Erbα/β.↑ PER2 protein	
	Tangeretin (Tan)	3					↑ Rhythmic activity of PER2 in PER2::Luc Clock Δ19/+ fibroblast cells	
	Naringin (NAR)	3–50 (Depends on experiment)					No Δ in the circadian rhythm of PER2 in either PER2::LucSV or mutant reporter cells.No binding to RORα/γ	
	Naringenin	3					No Δ in the circadian rhythm of PER2 in either PER2::LucSV or mutant reporter cells.	
Ribas-Latre et al. [91], 2015	Grape seed proanthocyanidin extract (GSPE)	N/A	Human HepG2	2-h Serum shock (50% horse serum)	Treatment with GSPE, collection every 3 h between 0 and 24 h time points. Control (ethanol)	RORα-LBD:Gal4-DBD reporter	↑ RORα activity. ↑ BMAL1 mRNA. Δ the acrophase of CLOCK, BMAL1, CRY1, RORα, and Rev-Erbα, but not PER2.↑ Rev-Erbα amplitude	
Kojima et al. [79], 2015	Biochanin A (BA), Genistein (GE), Formononetin (FN), and Daidzein (DA)	0.1–10	Chinese hamster CHO-K1, Human lymphoma T Jurkat cells and Mouse EL4	None	Treatment with various isoflavones. Control (0.01 M DMSO)	Rorα/γ:Luc reporter; RORα/γ-LBD transfected CHO-K1 cells; RORα/γ-knockdown EL4 cells	↑ RORα/γ activity and the interaction between LBD of both RORα/γ and LXXLL peptide, dose dependent, with varying levels of potency in CHO-K1 cells	↑ IL17A promoter in a dose-dependent manner in human lymphoma T Jurkat cells. ↑ dose of BA ↑ IL17A transcript in RORα and RORγ in EL4 cells
Dang et al. [90], 2015	Bavachalcone	2.5–20	Human HUVECs	2-h Serum shock (50% newborn bovine serum)	Treatment with bavachalcone/24 h. Control	N/A	↑ ROR-α1 mRNA and protein, dose-dependent (>2-fold and 6-fold at 20 μM, respectively). ↑ amplitude of BMAL1 at (28, 36, and 40 h)	
			Human HEK-293		Treatment with bavachalcone/16 h. Control	ROR-α1 (3× RORE) reporter luciferase plasmid construct	↑ ROR-α1 activity, dose-dependent (>3-fold at 20 μM)	
Parket al. [89], 2014	Resveratrol (Res)	10 and 100	Mouse NIH3T3	None	Treatment with Res/24 h.Control.	Ebox-Luc and PER1-Luc reporter combined with effector genes (CLOCK/BMAL1 and SIRT1)	↑ Dose ↓ PER1 activity under both basal and CLOCK/BMAL1-induced conditions in the presence of SIRT1.	
		100	Monkey COS-7		Treatment with Res/6 h. Control	Plasmids encoding SIRT1-VN:CLOCK-VC or SIRT1-VN:BMAL1-VC	Fail to affect the interaction between SIRT1 and CLOCK or BMAL1 domains	
Morioka. et al. [109], 2010	Genistein (GE)	50	Rat C6	Noradrenaline (NA) or Fsk (10 μM, 1 h)	Pretreatment gen/1 h and then NA or Fsk/1 h. Control.	N/A	↓ PER1 mRNA and protein.	
Oike and Kobori [102], 2008	Resveratrol (Res)	10–100	Rat-1 fibroblasts	None	Treatment with Res/8 h, collection at 0, 1, 2, 4, 8, and 14 h.Control (0.1% DMSO)	N/A	↑ BMAL1, PER1/2 mRNAs	

↑, Upregulation, improvement or high; ↓, downregulation or low; Δ, change.

ACC, acetyl-CoA carboxylase; ACR, acrylamide; AKT, protein kinase B; AML12, mouse hepatocyte; AMPK, adenosine monophosphate–activated protein kinase; BA, biochanin A; Bax/Bcl-2, apoptosis-related Bcl-2-associated X protein/ B-cell lymphoma 2; BDNF, brain-derived neurotrophic factor; BMAL1, brain and muscle ARNT-like 1; BMAL1-VC, BMAL1-C-terminal of Venus; BMSCs, human bone marrow-derived mesenchymal stem cells; BMP2, bone morphogenetic protein 2; BMSCs, human bone marrow-derived mesenchymal stem cells; BV2, mouse primary microglial cell lines; C6, Rat glioma cancer cells; C2C12, mouse skeletal myotube cells; CA, chicoric acid; CAP, capsaicin; CHO-K1, Chinese hamster ovary cells; CLOCK, circadian locomotor output cycles kaput; CLOCK-VC, CLOCK-C-terminal of Venus; COS-7, Monkey kidney fibroblast-like cells; COX6C, cytochrome c oxidase subunit 6C; CRY, cryptochrome; CT, circadian time; CUR, curcumin; DA, daidzein; DBD, DNA-binding domain; DBP, D-box binding PAR bZIP transcription factor; DEC1/2, differentiated embryonic chondrocyte-expressed gene 1/2; Dex, dexamethasone; dLuc, destabilized luciferase; EA, ellagic acid; EGCG, (– )-epigallocatechin-3-gallate; EL4, mouse lymphoma cells; Eluc, enhanced luciferase; FAS, fatty acid synthase; FBS, Fetal bovine serum; FFA, free fatty acid; FGF21, fibroblast growth factor 21; FN, formononetin; Fsk, forskolin; GE, genistein; GLUT2, glucose transporter 2; GSPE, grape seed proanthocyanidins extract; H1299, human lung carcinoma stem-like cells; HaCaT, human skin cell lines; HK-2, human kidney cells; HEK-293, human embryonic kidney 293 cells; HEK 293T, human embryonic kidney cells; HK-2, human kidney cells; Hepa1-6, murine hepatoma cell line; HepG2, human hepatocarcinoma cells; H/R, hypoxia and reoxygenation conditions; H_2_O_2_, hydrogen peroxide; HO-1, heme oxygenase-1; HUVECs, human umbilical vein endothelial cells; IL-6, interleukin-6; IL17A, interleukin 17A; iNOS, inducible nitric oxide synthase; L-02, derivative of human cervical cancer HeLa cells; LBD, ligand-binding domain; LS8, mouse ameloblast cell line; Luc, luciferase; MCF7, Human breast adenocarcinoma cells; MDA-MB-231, human breast adenocarcinoma cells; MMP, Mitochondrial membrane potential; mtDNA, mitochondrial DNA; mTOR, mammalian target of rapamycin; N/A, not applicable; NaF, sodium fluoride; NAR, naringin; ND, nondiabetic; NDUFS1, NADH:ubiquinone oxidoreductase core subunit S1; NIH3T3, embryonic mouse fibroblast cells; NOB, nobiletin; NPAS2, Neuronal PAS domain protein 2; NR1D1/2, nuclear receptor subfamily 1 group D member 1/2; Nrf2, nuclear factor erythroid 2-related factor 2; NQO1, NAD(P)H quinone oxidoreductase 1; OA, oleic acid; OPA1, optic atrophy protein 1; OXPHOS, oxidative phosphorylation; PA, palmitic acid; PBS, phosphate-buffered saline; p-CREB, phosphorylated cAMP response element–binding protein; PER, period; PGC-1α, peroxisome proliferator–activated receptor gamma coactivator 1-α; p-GSK-3β, phosphorylated glycogen synthase kinase-3 β; p-I-κB, phosphorylated nuclear factor kappa B; p-IRS-1, phosphorylated insulin receptor substrate-1; PM_2.5_, ambient particulate matter with a diameter of 2.5 μm; p-NF-κB, phosphorylated nuclear factor κ light chain enhancer of activated B cells; PPARα/γ, peroxisome proliferator–activated receptor α or γ; Que, quercetin; Res, resveratrol; Rev-Erbs, reverse erythroblastosis viruses; ROR, retinoic acid–related orphan receptors; RORE, ROR enhancer elements; ROS, reactive oxygen species; SH-SY5Y, human neuroblastoma cells; siRNA, small interference RNA; SIRT1, sirtuin1; SIRT6, sirtuin6; SIRT1-VN, SIRT1-Venus N-terminus-encoding plasmid; SREBP-1c, sterol regulatory element–binding protein-1c; T2D, type 2 diabetic; Tan, tangeretin; TGF-β1, transforming growth factor-β1; TNF-α, tumor necrosis factor; TP, tea polyphenols; U-2 OS, human osteosarcoma cells; ULT, urolithin A; A549, human lung carcinoma stem-like cells; 7,8-DHF, 7,8-dihydroxyflavone.

1 Only features of rhythmicity of clock genes quantified with analytical approaches of time-series analysis were considered.

2 Only the metabolic effects of polyphenols regulated by clock genes were presented.

3 Human adipose progenitor cells (APCs) derived from white-adipose tissue biopsies from abdominal subcutaneous region of young (age, 23.4 ± 2.1 y) and old females (age, 70.6 ± 5.9 y).

4 Human L-02 cells were identified in the original article as human normal liver cells.

5 Primary human myotubes derived from skeletal muscle biopsies obtained from normal glucose-tolerant donors.

6 Seven citrus flavonoid extracts include Fuju, Zijinougan, Daetiancheng, Jiweiputaoyou, Youliang, Manwengan, and Mabuwendan.

**FIGURE 3:**
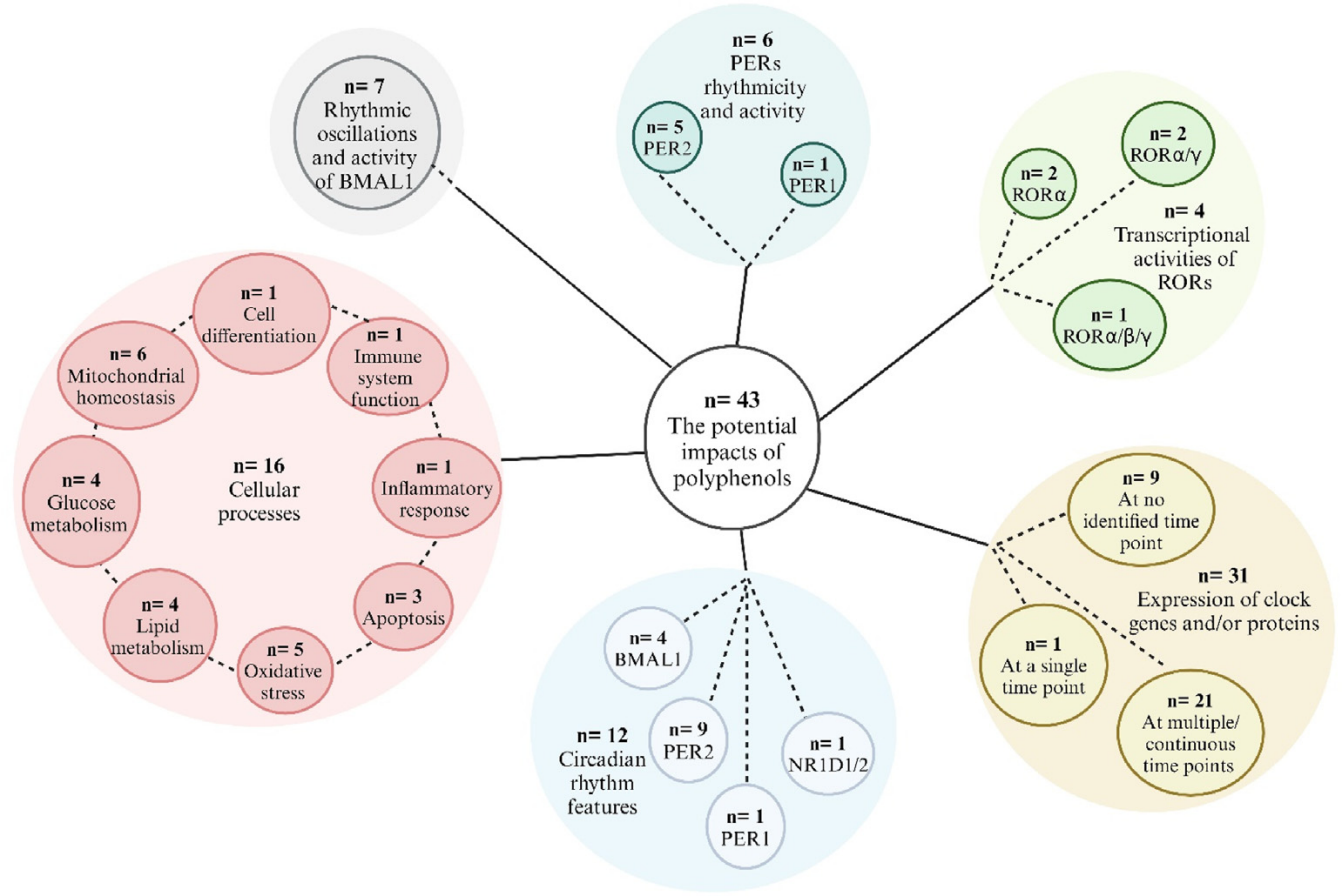
The distribution of polyphenols used in the included studies. *n*, number of studies. Created with https://www.biorender.com/.

**FIGURE 4:**
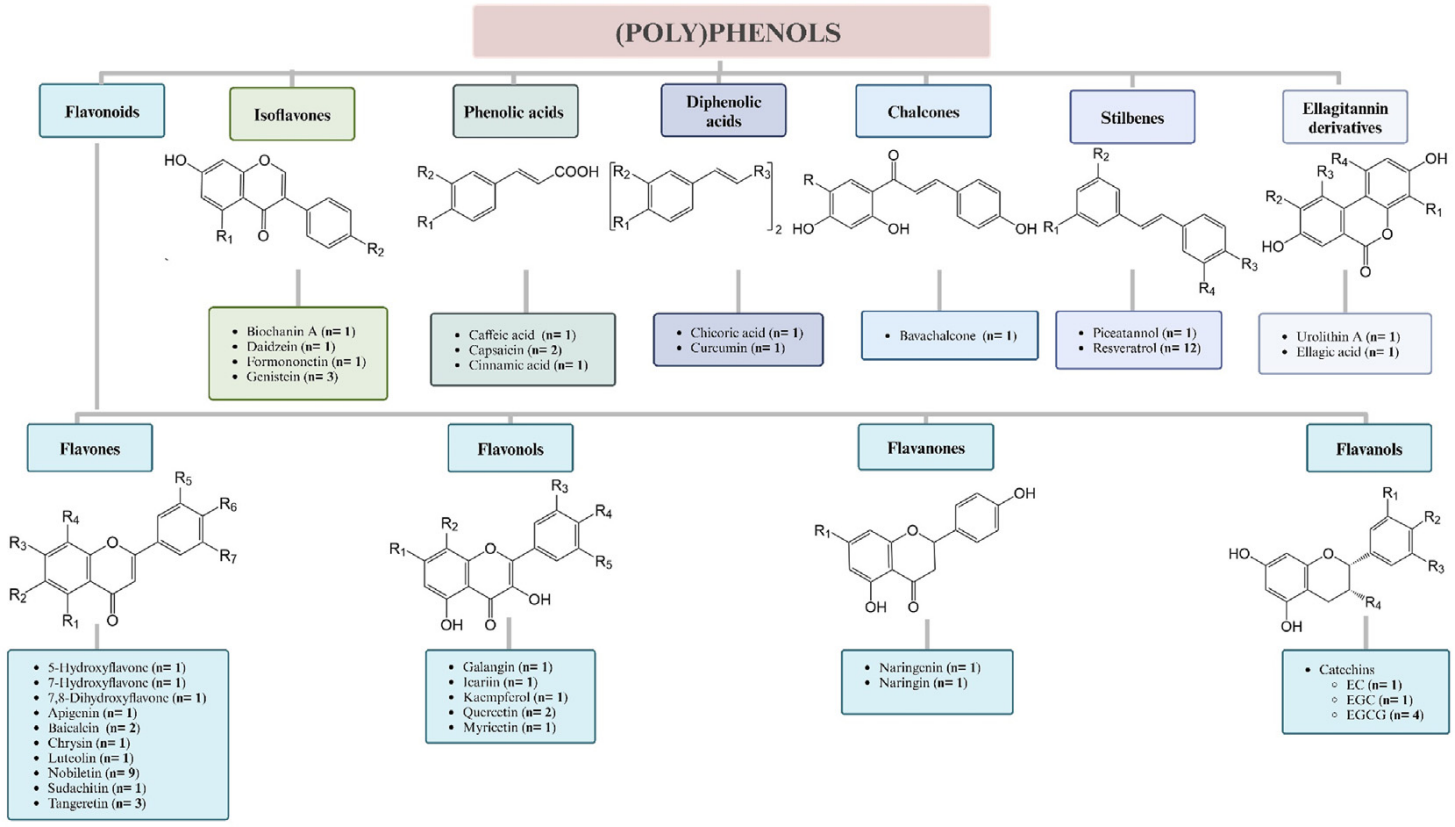
Classification and structure of polyphenols used in circadian studies in vitro. Abbreviations: EC, (– )-epicatechin; EGC, (– )-epigallocatechin; EGCG, (– )-epigallocatechin-3-gallate; *n,* number of studies; R groups substituted as indicated below. No information was found on anthocyanidins nor lignans. 5-Hydroxyflavone: R1= OH; R2= R3= R4= R5= R6= R7= H; 7-hydroxyflavone: R1= R2= R4= R5= R6= R7= H; R3= OH; 7,8-dihydroxyflavone: R1= R2= R5= R6= R7= H; R3= R4= OH; apigenin: R1= R3= R6= OH; R2= R4= R5= R7= H; baicalein: R1= R2= R3= OH; R4= R5= R6= R7= H; bavachalcone: R1= CH_2_CH=CH(CH_3_)_2_; biochanin: R1= OH; R2= OCH_3_; caffeic acid: R1= R2= OH; capsaicin: R1= OH; R2= OCH_3_; –COOH replaced by: –NHC(=O)CH_2_CH_2_CH_2_CH_2_CH=CHC(CH_3_)_2_; chrysin: R1= R3= OH; R2= R4= R5= R6= R7= H; chicoric acid: R1= R2= OH; R3= –C(=O)OC(C(=O)OH)C((=O)OH)OC(=O)–; cinnamic acid: R1= R2= H; curcumin: R1= OH; R2= OCH_3_; R3= –C(=O)CC(=O)–; daidzein: R1= H; R2= OH; EC: R1= H; R2= R3= R4= OH; EGC: R1= R2= R3= R4= OH; EGCG: R1= R2= R3= OH; R4=gallic acid; ellagic acid: R1= R2= OH; R3 and R4= R3-O(C=O)–R4 ; formononetin: R1= H; R2= OCH_3_; galangin: R1= OH; R2= R3= R4= R5= H; genistein: R1= R2= OH; icariin: R1= glucose; R2= CH_2_CH=CH(CH_3_)_2_; R3= rhamnose; R4= OCH3; R5= H; kaempferol: R1= R4= OH; R2= R3= R5= H; luteolin: R1= R3= R6= R7= OH; R2= R4= R5= H; myricetin: R1= R3= R4= R5= OH; R2= H; naringenin: R1= H; naringin: R1= glucorhamnoside; nobiletin: R1= R2= R3= R4= R6= R7= OCH_3_; R5= H; piceatannol: R1= R2= R3= R4= OH; quercetin: R1= R3= R4= OH; R2= R5= H; resveratrol: R1= R2= R3= OH; R4= H; tangeretin: R1= R2= R3= R4= R6= O; R5= R7= H; urolithin A: R1= R2= R3= R4= H. Created with https://www.biorender.com/

From the 43 studies, a total of 34 different (poly)phenols were studied for their impact on clock-mediated cellular processes (*n* = 16 papers), clock gene and/or protein expression (*n* = 31), specifically focusing on the rhythmicity and activity of BMAL1 (*n* = 7), PERs (*n* = 6) and RORs (*n* = 4), or circadian rhythm characteristics of clock genes (*n* = 12), as shown in Figure 3. Resveratrol was the most commonly studied, followed by nobiletin; (–)-epigallocatechin-3-gallate (EGCG); tangeretin and genistein; and capsaicin, quercetin, and baicalein (Figure 4). In addition, 6 studies identified the effect of (poly)phenol-rich extracts or mixtures, which include citrus fruit extract (*n* = 1), grape seed proanthocyanidin extracts (*n* = 2), *Quercus gilva* extract (*n* = 1), and tea (poly)phenols (*n* = 2). There were 18 studies with no cell synchronization and 22 studies with cell synchronization, which deployed a 2-h serum shock (*n* = 16), Dex (*n* = 10), or Fsk (*n* = 6). Across all studies, 25 used human cells and 24 employed animal cells.

### Synthesis of results

#### Circadian-dependent effects of (poly)phenols on metabolism and other pathways

A total of 16 studies considered the potential clock-mediated impact of (poly)phenols on diverse cellular processes, such as metabolic homeostasis (*n* = 13), which encompassed mitochondrial homeostasis (*n* = 6), glucose metabolism (*n* = 4), and lipid metabolism (*n* = 4), followed by studies focusing on oxidative stress (*n* = 5), apoptosis (*n* = 3), inflammatory response (*n* = 1), immune system function (*n* = 1), and cell differentiation (*n* = 1).

Six studies addressed the influence of (poly)phenols on mitochondrial function in relation to circadian clock genes, mainly *BMAL1*. In both immortalized (human hepatocarcinoma HepG2) and primary (cultured mouse hepatocytes) cells, capsaicin, resveratrol, and tea (poly)phenols attenuated mitochondrial dysfunction. This was achieved by alleviating the loss of mitochondrial membrane potential with 50 μM capsaicin [67,68] and by enhancing the protein expression of all mitochondrial respiration complexes, along with transcripts involved in oxidative phosphorylation, such as NADH:ubiquinone oxidoreductase core subunit S1 (*NDUFS1*) and cytochrome c oxidase subunit 6C (*COX6C*) with 50 μM resveratrol and with 10 and 40 μg/mL tea (poly)phenols [69,70]. Concomitantly, it was reported that resveratrol resulted in an increase in the transcripts of mitochondrial biogenesis, including *SIRT1* and *PGC-1α*, as well as SIRT1 protein, whereas tea (poly)phenols upregulated transcripts of specific markers related to mitochondrial dynamics and restored the shallow circadian oscillation of the SIRT1 protein [69,70]. Similar to those findings observed in tea (poly)phenol-treated hepatic cells, human neuroblastoma SH-SY5Y cells treated with tea (poly)phenols at a concentration of 40 μg/mL showed restorative effects on SIRT1 and mitochondrial complex proteins [71]. In support, a study on human kidney HK-2 cells treated with a similar concentration of resveratrol improved disruptions caused by BMAL1 knockdown, by augmenting mitochondrial membrane potential and mitochondrial DNA quantity via upregulation of mitochondrial biogenesis SIRT1/PGC-1α signaling [72]. It must be noted that the positive effects of those (poly)phenols was elucidated in both synchronized and nonsynchronized cells, as well as in a *BMAL1*-dependent manner.

**FIGURE 5:**
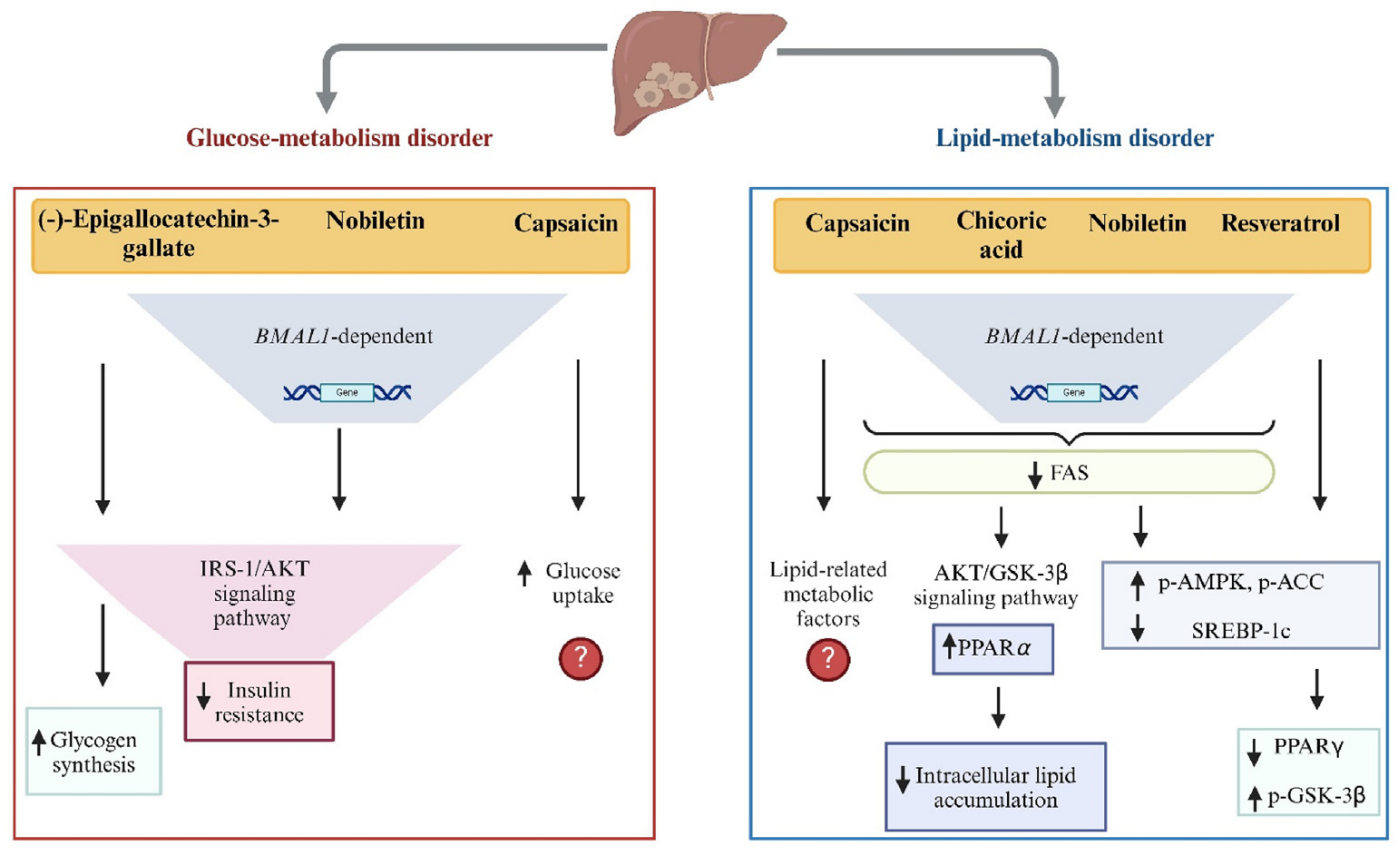
The purported impact of polyphenols on clock-mediated hepatic glucose and lipid metabolism disorders, based on effects observed in human hepatocarcinoma HepG2 cells or primary mouse hepatocytes. Abbreviations: ↑, increase; ↓ decrease; ?, a mechanism not proposed; BMAL1, brain and muscle ARNT-like 1; AKT, protein kinase B; FAS, fatty acid synthase; GSK-3β, glycogen synthase kinase-3 β; IRS-1, insulin receptor substrate-1; p-ACC, phosphorylated acetyl-CoA carboxylase; p-AMPK, phosphorylated adenosine monophosphate–activated protein kinase; p-GSK-3β, phosphorylated glycogen synthase kinase-3 β; PPARα/γ, peroxisome proliferator–activated receptor α or γ; SREBP-1c, sterol regulatory element–binding protein-1c. Created with https://www.biorender.com/.

Five studies examined the role of (poly)phenols against oxidative imbalance in cells transfected with *BMAL1* small interference RNA (si-*BMAL1*). Capsaicin and resveratrol, both administered at a concentration of 50 μM in nonsynchronized HepG2 cells, effectively alleviated cellular oxidative stress through suppressing the production of reactive oxygen species and boosting the nuclear factor erythroid 2-related factor 2 (Nrf2)/NAD(P)H quinone oxidoreductase 1 (NQO1) pathway in a *BMAL1*-dependent manner, respectively [[67], [68], [69]]. Similar to resveratrol, in the presence of *BMAL1*, tea (poly)phenols protected HepG2 cells, nonsynchronized primary mouse hepatocytes and human neuroblastoma SH-SY5Y cells from H_2_O_2_-induced oxidative stress, promoting antioxidant defense mechanisms [70,71].

(Poly)phenols were also reported to modulate disrupted glucose metabolism and insulin resistance via *BMAL1* involvement, as evidenced by 4 studies (Figure 5). Nobiletin administered at a high concentration (200 μM) to primary cultured mouse hepatocytes caused an increase in phosphorylated insulin receptor substrate-1 (p-IRS-1), thereby activating its downstream targets, including the phosphorylation of protein kinase B (AKT), glycogen synthase kinase-3β (GSK-3β), and cAMP response element–binding protein (CREB), as well as glucose transporter 2 (GLUT2) membrane translocation, in the presence of *BMAL1*. This activation alleviated glucose metabolic disorder via insulin signaling pathways [73]. The same effect was seen in primary cultured mouse hepatocytes and HepG2 cells treated with EGCG at concentrations of 10 and 50 μM, respectively, in addition to its role in stimulating glycogen synthesis [74]. In human pancreatic type 2 diabetes islet cells triggered by glucose, insulin secretion was improved in response to 20 μM nobiletin, irrespective of *CLOCK* deficit [75]. A study on capsaicin demonstrated that, at a concentration of 50 μM, it enhanced hepatic glucose uptake, although notably the HepG2 cells were not synchronized [68].

Additionally, 4 studies reported the effect of (poly)phenols on hepatic lipid disorders with respect to circadian clock genes in either HepG2 cells or primary mouse hepatocytes, summarized in Figure 5. Upon treatment with high concentrations of capsaicin, resveratrol, nobiletin, and chicoric acid (50, 100, 200, and 200 μM, respectively), lipid disturbance was attenuated in a *BMAL1*-dependent manner, with a decrease in the protein responsible for hepatic lipid synthesis, fatty acid synthase (FAS) [67,73,76,77]. Despite the use of unsynchronized HepG2 cells, capsaicin treatment reduced the expression of lipogenic proteins, including acetyl-CoA carboxylase (ACC), mammalian target of rapamycin (mTOR), and PGC-1α, in contrast to that of AKT and AMPK [67]. Resveratrol and nobiletin stimulated the phosphorylation of ACC via AMPK activation and diminished key enzyme concentrations of de novo lipogenesis, such as sterol regulatory element–binding protein-1c (SREBP-1c) [73,76]. Chicoric acid restored disrupted lipid metabolism under high fat conditions by mediating the AKT/GSK-3β signaling pathway and preventing intracellular lipid accumulation. Of note, it also decreased SIRT1 and PGC-1α proteins in the absence of circadian clock regulators, following treatment with si-*BMAL1* and si-*CLOCK* [77]. Unlike resveratrol, which downregulated peroxisome proliferator–activated receptor γ (PPARγ) [76], chicoric acid upregulated *PPARα*, responsible for restoring lipid homeostasis [77].

To assess the efficacy of (poly)phenols in preventing apoptosis through clock genes, 3 studies showed that resveratrol, EGCG, and tea (poly)phenols exerted protective effects when *BMAL1* was silenced. In nonsynchronized human kidney HK-2 cells, pretreatment with 50 μM resveratrol recovered the perturbations caused by *BMAL1* knockdown, decreasing the percentage of apoptosis and concentrations of apoptosis-related proteins, cytoplasmic cytochrome c, and cleaved caspase-3. The findings suggest that its effect is mediated by mitochondrial homeostasis [72]. The same favorable effect was seen in rat nucleus pulposus cells (NPCs), where 20 μM EGCG lowered the apoptotic protein cleaved caspase-3 and the apoptosis-related Bcl-2-associated X protein/ B-cell lymphoma 2 (Bax/Bcl-2) ratio in the presence of *BMAL1* [78], with Bcl-2 regarded as an antiapoptotic protein. Furthermore, another study reported that pretreatment of tea (poly)phenols at a concentration of 40 μg/mL was neuroprotective, safeguarding neurons from apoptotic cell death and promoting their survival via activation of the AKT/CREB/BDNF signaling cascade in human neuroblastoma SH-SY5Y cells [71]. However, it is unlikely that tea (poly)phenols will reach neurons because they are metabolized by endogenous enzymes and/or the gut microbiota before reaching the blood.

In a single study, the anti-inflammatory properties of resveratrol were investigated in acrylamide-induced unsynchonized HepG2 cells. Pretreatment with resveratrol at a very high concentration (50 μM) improved inflammatory responses via the inflammation signaling pathway, by inactivating phosphorylation of nuclear factor κ light chain enhancer of activated B cells (NF-κB) and inhibitor of nuclear factor kappa B (I-κB), and dampening the mRNA of pro-inflammatory cytokines, including tumor necrosis factor (TNF-α), inducible nitric oxide synthase (iNOS), and interleukin-6 (IL-6), in a *CRY1*-dependent manner [69]. However, none of these effects are likely to be physiologically relevant given the high concentration of resveratrol used.

One study reported the effect of 4 isoflavones, namely, biochanin A, genistein, formononetin, and daidzein, on modulating the immune response via *RORα/γ* in nonsynchronized cells. Through RORα/γ, all isoflavones (0.1–10 μM) exhibited a dose-dependent activation of the interleukin 17A (IL17A) promoter in human lymphoma T Jurkat cells with different potencies, with biochanin A being most effective. This promoter is responsible for initiating transcription and promoting the production of IL-17A protein, which plays a crucial role in inflammatory and autoimmune disorders. In *RORα* and *RORγ*-knockdown mouse lymphoma EL4 cells, biochanin A (10 μM) downregulated *IL17A* mRNA expression [79].

In terms of cell differentiation, one study assessed the association between icariin and *BMAL1* in osteogenic differentiation of human bone marrow–derived mesenchymal stem cells (BMSCs). In a *BMAL1*-dependent manner, the application of 100 μM icariin upregulated the expression of mRNA and proteins involved in bone formation, including bone morphogenetic protein 2 (BMP2), RUNX family transcription factor 2, alkaline phosphatase, and osteocalcin (OC). Additionally, parallel effects were observed in BMSCs with *BMAL1* overexpression. It was suggested that icariin has a potential effect on bone mineralization by promoting osteogenic differentiation through BMAL1-BMP2 signaling, making it a possible therapeutic agent for osteogenic disorders [80]. Notably, this action was observed without synchronization of the cells and an extremely high concentration of icariin, and so is of doubtful physiological relevance.

#### BMAL1 rhythmicity and activity

Seven studies monitored the rhythmic oscillations of *BMAL1* for 4–7 continuous days in various *BMAL1*-luciferase (*BMAL1*-Luc) reporter knock-in cells. Several (poly)phenols, including nobiletin (*n* = 4 papers), tangeretin (*n* = 1), sudachitin (*n* = 1), and *Quercus gilva* extracts (*n* = 1), resulted in consistent effects on *BMAL1* oscillations at various concentrations in a range of cell types. In both human bone osteosarcoma U-2 OS cells, characterized by robust circadian patterns, and human breast adenocarcinoma MCF7 cells, with weaker oscillations, nobiletin concentrations of 5 and 50 μM showed consistent yet subtle effects on oscillations. In contrast, the rhythmicity of *BMAL1* in human breast adenocarcinoma MDA-MB-231 cells, known for their arrhythmic nature, was improved with a high concentration (50 μM) of nobiletin [81]. Comparable oscillations were observed in human pancreatic islets isolated from donors with type 2 diabetes treated with 20 μM nobiletin [75]. A range of nobiletin concentrations (0.3–10 μM) activated *BMAL1* promoter–driven luciferase expression in an *RORα/γ*-dependent manner in both synchronized murine hepatoma Hepa1-6 cells and U-2 OS cells [82], and nobiletin (10 μM) elicited robust rhythmic oscillations of *BMAL1*-Luc in mouse 3T3-L1 preadipocyte cells [83]. The effect of tangeretin enhanced both *BMAL1*-Luc activity and oscillation dose dependently (0.1–5 μM) in RORα/γ-overexpressing U-2 OS cells and in unsynchronized embryonic mouse fibroblast NIH3T3 cells [84]. Administration of sudachitin, a citrus polymethoxyflavone, induced rhythmic oscillations of *BMAL1*-Luc in a dose-dependent manner (1–10 μM) in synchronized human bone osteosarcoma U-2 OS cells [85]. A *Quercus gilva* ethanol extract dose dependently increased the rhythmic expression of *BMAL1* [86].

#### PERs rhythmicity and activity

Six studies recorded the rhythms of *PERs* for 4–7 d, of which 5 focused on *PER2* and only 1 on *PER1,* using luciferase-expressing reporter cells. Exposure of mouse microglia BV-2 cells to 7 citrus flavonoid extracts stimulated *PER2* expression and the findings suggested that an enhanced oscillation of PER2 and inhibitory effect on LPS-induced circadian disruption of PER2 expression were specifically correlated to the presence of polymethoxyflavones with 4 or more methoxy groups, namely, nobiletin and tangeretin [87]. Consistent with this, tangeretin (3 μM) increased the rhythmic activity of *PER2* in *PER2*::Luc Clock^Δ19/+^ fibroblast cells; however, the analogs naringin and naringenin showed no effect on the circadian rhythm of *PER2* in either *PER2*::LucSV or mutant reporter cells [82]. Similarly, nobiletin at concentrations of 5 and 50 μM exhibited modest yet consistent rhythmic patterns for *PER2* in both human bone osteosarcoma U-2 OS cells and human breast adenocarcinoma MCF7 cells. The higher concentration expressed a noticeable improvement in *PER2* rhythmicity in human breast adenocarcinoma MDA-MB-231 cells [81]. Moderate effects of sudachitin (1–10 μM) on *PER2* oscillations were reported in *PER2*::LucSV mouse fibroblast cells, in agreement with the findings of nobiletin in U-2 OS cells and MCF7 cells [85]. Furthermore, a rhythmic pattern of *mPER2* in rat C6 cells glioma cancer cells was induced by 5 μM curcumin 88]. An important point to note is that various cell synchronization techniques were applied in the aforementioned studies, increasing the potential physiological relevance.

About the transcriptional activity of *PER1*, nonsynchronized mouse embryonic fibroblast (MEF) (NIH3T3) cells, co-transfected with a *PER1*-Luc reporter in combination with or without *CLOCK*/*BMAL1* and *SIRT1,* were treated with resveratrol concentrations of 10 or 100 μM. The study revealed that high concentrations significantly downregulated *PER1* activity under both basal and *CLOCK/BMAL1*-induced conditions, indicating that changes in gene suppression could be ascribed to the activation of *SIRT1* by resveratrol [89].

#### RORs activity

Among the studies reviewed, 4 investigated the possible impact of polyphenols on the transcriptional activities of *RORs*, consisting of a combination of α, β, and γ isoforms: *RORα* (*n* = 2 papers), *RORα/γ* (*n* = 2), and *RORα/β/γ* (*n* = 1).

In unsynchronized human embryonic kidney HEK 293T cells, both nobiletin and tangeretin exhibited substantial inhibition of *RORα* and *RORγ* at a low concentration (5 μM), but no effects were seen on *RORβ* activity. Furthermore, tangeretin strongly and dose dependently reduced *ROR*α/γ activity, with half-maximal inhibitory concentrations (IC_50_) of 0.67 and 0.39 μM, respectively. The findings suggest that tangeretin is a *ROR* agonist, thereby indirectly promoting the expression of the target gene *BMAL1* by interacting with these receptors [84]. Similarly, nobiletin, acting as a ROR agonist, directly bound to *ROR*α/γ in HEK 293T cells, whereas naringin did not bind to *ROR*α/γ in Hepa1-6 cells [82]. Biochanin A, genistein, formononetin, and daidzein enhanced *RORα* and *RORγ* activities in a dose dependently (0.1–10 μM), with varying levels of potency, with biochanin A and formononetin being notably more potent in unsynchronized Chinese hamster ovary CHO Tet-on cells. Likewise, the interactions between the ligand-binding domain of both RORα/γ and the LXXLL peptide of their coactivators in unsynchronized CHO-K1 cells was dose dependently strengthened by all isoflavones [79]. In the same way, *RORα* activity was increased with an increasing dose of bavachalcone in HEK-293 cells, by >3-fold at 20 μM [90]. Grape seed proanthocyanidin extract (100 mg/L) also caused a significant augmentation in *RORα* activity, which positively affects the expression of BMAL1, in HepG2 cells [91]). However, the proanthocyanidins in such an extract would be metabolized by endogenous enzymes and/or the gut microbiota before reaching the liver in vivo.

#### (Poly)phenol regulation of clock gene and/or protein expression

We identified 31 studies in which the expression of clock genes and/or proteins was monitored. Of these, expression was measured at no identified time point (*n* = 9), at a single point (*n* = 1), or at multiple/continuous time points (*n* = 21).

Nine studies investigated the influence of (poly)phenols on mRNA and/or protein concentrations with, remarkably, no identified temporal reference and in the absence of cell synchronization across virtually all these publications. The impacts of cardiac fibrosis–causing transforming growth factor-β1 (TGF-β1) on circadian clock proteins was investigated in neonatal primary mouse cardiac fibroblast (CF) cells, whereas concurrent treatment with 7,8-dihydroxyflavone (10 μM) markedly lowered BMAL1, PER2, and CRY2 protein concentrations. It was postulated that 7,8-dihydroxyflavone serves as an antifibrotic agent through modulating circadian rhythmic-mediated signals [92]. Another finding is that a significant dose-dependent reduction in CLOCK, at both the gene and protein concentrations, was observed in human lung carcinoma stem-like A549 and H1299 cells in response to high concentrations of EGCG (20 and 40 μM) [93]. Pretreatment with 50 μM resveratrol in sodium fluoride-exposed mouse ameloblast LS8 cells resulted in an augmentation of CLOCK, BMAL1, and PER2 proteins [94].

The effects of resveratrol, quercetin, and caffeic acid were assessed on human lung fibroblast cells of different age groups (TIG-1-20, young cells; and TIG-1-60, old cells) with outcomes dependent on the age of the cells and concentration of (poly)phenol applied. Both quercetin and resveratrol (at concentrations of 2 and 100 μM, respectively) significantly enhanced *BMAL1* mRNA in both types of fibroblast cells. Although increasing *NR1D1* and *SIRT1* coupled with decreasing *PER1* and *SIRT6* transcript concentrations in young cells, resveratrol led to a rise in *PER1* and *SIRT6* concentrations and a decline in *SIRT1* within old cells. Unlike the effect of the higher dose of quercetin (20 μM) in young fibroblast cells on *SIRT1*, quercetin concentrations of (2 and 100 μM) demonstrated upregulation of *SIRT6* and *NR1D1*. However, both doses of quercetin, in particular the higher one, reduced *PER1* expression in young cells, which contrasts with the effect in old cells. Conversely, caffeic acid (at a low concentration of 2.5 μM) promoted the gene expression of *PER1* in both young and old fibroblast cells. A reduction in *BMAL1* and *SIRT1* levels with caffeic acid in an inverse dose–response in young cells was observed, whereas boosting only *BMAL1* concentrations in a dose–response manner in old fibroblast cells. In addition, in both young and old fibroblast cells, *SIRT6* transcript was increased by 1.6- and 2.5-fold, respectively, in response to the higher concentration of caffeic acid (25 μM). Interestingly, a dramatic elevation in BMAL1 protein was induced by the 3 (poly)phenols in comparison with the control in young fibroblast cells; nevertheless, only resveratrol did not affect BMAL1 protein in aged cells. Meanwhile, young fibroblast cells treated with lower doses of quercetin and caffeic acid demonstrated a significant enhancement in PER1 protein, and together, these results indicate that these 3 (poly)phenols exerted regulatory effects on different circadian genes through diverse mechanisms [95].

Additionally, baicalein at a concentration of 10 μM demonstrated a downregulatory effect on *BMAL1* in *RORα*-silenced C2C12 mouse skeletal myotubes [96]. It was also found that icariin, administrated at a low concentration of 0.1 μM, enhanced both the mRNA and protein concentrations of BMAL1 in human BMSCs, thereby facilitating osteogenic differentiation via BMAL1 [80]. Because icariin is a prenylated flavonol glycoside, it is unlikely to be absorbed intact into the blood. Compared with the upregulation of *BMAL1* transcript by 5 μM tangeretin in synchronized mouse primary microglial BV2 cells, tangeretin enhanced rhythmic gene expression of *BMAL1* and *PER2* in a dose-dependent manner (0.1, 1, and 5 μM) in nonsynchronized embryonic mouse fibroblast NIH3T3 cells [84]. Pretreatment of nonsynchronized cardiomyocyte cells isolated from young Sprague–Dawley rats with 25 μM nobiletin revealed a substantial increase in RORα protein concentrations, suggesting a restorative impact of nobiletin on cardiac cell autophagy and survival under hypoxia-induced stress via RORα induction [97].

One study evaluated the expression of neuronal PAS domain protein 2 (*NPAS2*), a forebrain-localized paralog of *CLOCK*, at a single time point, specifically 28 h after synchronization in primary human myotubes derived from skeletal muscle biopsies obtained from normal glucose-tolerant donors. Resveratrol (10 μM) attenuated the expression of *NPAS2*, which was increased by the deletion of optic atrophy protein 1 (*OPA1*), crucial for normal mitochondrial morphology and function [98].

If we consider the 21 studies analyzing the expression of clock genes and proteins at multiple or continuous time points, significant points to note within those studies include discrepancies observed in cell synchronization techniques, treatment conditions conducted either in the presence or absence of various metabolic stressors, the timing of treatments, and timing of cell sample collection. Most studies reported the use of synchronized cells. As an example, 2 different concentrations of grape seed proanthocyanidin extract (25 and 100 mg/L) exerted opposite effects on the *BMAL1* transcript in synchronized HepG2 cells, with the lower dose downregulating *BMAL1* at 12 h and the higher dose upregulating *BMAL1* at 1 and 15 h [91,99]. However, it is unlikely that such high concentrations of proanthocyanidins would reach internal organs such as the liver, because they are catabolized by the gut microbiota before absorption in lower molecular weight phenols. A range of cinnamic acid concentrations (0.01–10 μM) caused no increase in the expression of *BMAL1*, *PER1/2*, and *Rev-Erbα* compared with the control in synchronized neuronal cells obtained from C57BL/6J mice [100]. In synchronized mouse fibroblast cells, 5 μM nobiletin altered transcript levels of *CLOCK*, *BMAL1*, *NPAS2*, *PER2*, *CRY1/2*, *RORα/γ*, and *Rev-Erbα/β* and increased PER2 protein [82]. In contrast, nobiletin concentrations of 10 and 20 μM resulted in a dose-dependent enhancement of the gene expression of *BMAL1* and *CRY1* in synchronized mouse 3T3-L1 preadipocyte cells, offering insights into the potential management of obesity-related diseases at a cellular level [83]. Upon exposure to a concentration of 10 μM sudachitin, human U-2 OS showed upregulation of BMAL1, PER2, and CRY1 at the gene and protein levels*,* along with downregulation of Rev-Erbα gene and protein [85].

Similarly, 5 studies explored the effects of resveratrol on circadian clock genes, but all used extremely high concentrations. Resveratrol (50 and 100 μM) treatment of 2 distinct cell types, synchronized mouse hepatocyte AML12 cells and nonsynchronized rat-1 fibroblast cells, respectively, enhanced the transcription of *BMAL1* [101,102]. In addition, in rat-1 fibroblast cells, both *PER1* and *PER2* genes were upregulated in response to resveratrol at 100 μM [102]. In the case of metabolically stressed cells, both synchronized HepG2 cells and primary mouse hepatocytes, when pretreated with resveratrol (100 μM), displayed comparable patterns with those in nonstressed cells. There was an augmentation in the expression of clock genes and proteins in HepG2, including CLOCK, BMAL1, PER1/2, CRY1, and Rev-Erbα [76], and in increase in protein expression of CLOCK, BMAL1, SIRT1, and CRY1 in primary mouse hepatocytes [69]. To compare its impacts within synchronized human adipose progenitor cells (APCs) of old and young female groups, treatment with 100 μM resveratrol caused fluctuating expression patterns of *CRY1* and *NR1D1* across time, along with enhanced *SIRT1* in both types of cells. It promoted the expression of various clock genes, *CRY2*, *NR1D2*, and *RORα,* specifically in young cells. However, the upregulated transcript levels of *BMAL1* at multiple time points were exclusively observed in the older cells, with an opposing effect on *CLOCK*. The authors postulated a putative role of resveratrol in the aging process by targeting clock components [103]. It is of note that resveratrol was the most frequently utilized polyphenol in these studies, particularly at very high concentrations, despite its trace amount in natural sources and poor bioavailability in its original form [110]. This poses a challenge in translating its biological effects in humans.

To further examine the role of (poly)phenols in modulating circadian clock oscillators under metabolic disorder conditions, 10 studies demonstrated some restorative effects of various (poly)phenols. Some (poly)phenols were applied to cells prior to the metabolic stressor, whereas some were cotreated with the metabolic stressor, with stressors used including glucosamine, H_2_O_2_, LPS, free fatty acids, or ambient particulate matter (PM). Pretreatment with 20 μM EGCG in rat nucleus pulposus cells (NPCs) and cotreatment with EGCG in HepG2 cells (50 μM) and in primary mouse hepatocytes (10 μM), all of which were synchronized, recovered shallow oscillations of clock genes, involving *BMAL1*, *CLOCK*, *PER1/2*, and *CRY1/2*, as well as BMAL1 protein expression levels [74,78]. In primary mouse hepatocytes, the protein concentrations of CLOCK and SIRT1 were also elevated [74]. As such, polymethoxyflavones, specifically containing 4 or more methoxy groups, enhanced the expression of *CLOCK*, *BMAL1*, *PER1/2/3*, *CRY1/2*, *Rev-Erbα/β*, *RORα*, and *NPAS2* circadian genes in synchronized mouse microglia BV-2 cells pretreated with either citrus flavonoid extracts or nobiletin [87]. In the same way, pretreatment with nobiletin at very high concentration (200 μM) amplified the shallow mRNA fluctuations of clock oscillators *CLOCK*, *BMAL1*, *PER2*, *CRY2*, and *Rev-Erbα* in synchronized primary mouse hepatocytes, while also enhancing the protein expression of CLOCK and BMAL1 in nonsynchronized HepG2 [73]. Likewise, when synchronized cells such as HepG2 and human neuroblastoma SH-SY5Y cells, were pretreated with the same concentration of tea (poly)phenols (40 μg/mL), the circadian disruption of clock components at both the gene level (*CLOCK*, *BMAL1*, *PER1/2*, *CRY1/2*) and protein level (CLOCK, BMAL1), as well as SIRT1 protein, was restored [70,71]. In a comparable way to tea (poly)phenols and EGCG, cotreatment with chicoric acid at a high concentration of 200 μM also improved the subtle oscillations of transcript levels for all clock genes, along with *Rev-Erbα,* in synchronized HepG2 cells [77]. In nonsynchronized HepG2 cells, a combined treatment with capsaicin at a concentration of 50 μM alleviated the disruption at both clock gene and protein concentrations of CLOCK, BMAL1, PER1/2, CRY1/2, and Rev-Erbα [67,68]. Collectively, these studies support the notion that (poly)phenols possess the potential to effectively restore circadian rhythm disruption, particularly in a metabolic disorder context.

In a single study, the impacts of a combined treatment involving resveratrol (50 μM) and ambient particulate matter (PM), an environmental pollutant associated with disrupted glucose metabolism, were examined on circadian clock proteins in unsynchronized PM_2.5_-exposed human L-02 cells. Resveratrol restored the rhythmic expression of BMAL1 and CLOCK proteins, as well as SIRT1 protein [104]. Human L-02 cells were mistakenly identified in this context as human normal hepatocytes for investigating hepatic glucose metabolism, but in fact, these cells are derived from the HeLa human cervical cancer cell line [111]. In light of this misidentification, results should be interpreted with caution.

#### Role of (poly)phenols on the circadian rhythm characteristics of clock genes

Twelve studies calculated cardinal circadian rhythm parameters of clock genes, mainly *PER2* (*n* = 9), *BMAL1* (*n* = 4), *PER1* (*n* = 1), and *NR1D1/2* (*n* = 1), in response to diverse (poly)phenols under different treatment strategies in various synchronized cells, utilizing a range of analytical approaches.

Chronic treatment of MEFs with lower concentrations of urolithin A, a microbial metabolite of ellagitannins, dose dependently extended the period of *PER2*::Luc (at 10 or 20 μM) and *BMAL1*-enhanced luciferase (Eluc) (at 10 μM), along with an enhanced amplitude. Conversely, the highest concentration of ellagic acid (100 μM) prolonged the period and decreased the amplitude of *PER2*. Upon acute treatment for 30 min, both urolithin A and ellagic acid resulted in a phase delay (the time at which the peak or trough in the circadian cycle delays) of *PER2* in a dose- and time-dependent manner [105]. Similarly, when exposing the same cells to continuous treatment with 18 different flavonoids, in an attempt to further examine how their structural differences affected circadian features, each displayed distinct dose-dependent impacts on the period and amplitude of *PER2*, with 7-hydroxyflavone being the only flavonoid to cause a delayed first peak at 10 μM. Most importantly, among the tested flavonoids, only nobiletin and tangeretin enhanced the amplitude and extended the period, showing positive correlations between amplitude and period. However, under transient treatment conditions, flavones, flavonols, isoflavones, and polymethoxyflavones, excluding catechins, elicited a dose-dependent phase delay of *PER2* [106]. In MEFs, the transient application of passion fruit seed extract and piceatannol at a concentration of 100 μM, at 2 different timing intervals (CT4 and CT20), contributed to an increased amplitude of *PER2*::Luc. These treatments also time dependently affected the peak phase, triggering a phase advance (the time at which the peak or trough in the circadian cycle advances) specifically at CT4 [107].

Additionally, another study reported that nobiletin concentrations of 5 and 50 μM gave contrasting effects on the circadian characteristics of *BMAL1* and *PER2* across different cultured cell models in terms of their baseline rhythmicity. In human bone osteosarcoma U-2 OS cells, both doses slightly prolonged periods of *BMAL1* and *PER2*, with greater effects observed with the higher concentration, whereas the damping rate (at which the amplitude of a circadian rhythm decreases over time) of *PER2*::Luc oscillations was changed by the lower concentration. In contrast, both concentrations decreased the amplitude of *BMAL1* and *PER2* in human breast adenocarcinoma MCF7 cells, with a variety of effects on period, amplitude, and damping rate across treatments depending on the estimation technique used, as opposed to the consistent effects noticed in the former cells [81]. However, a range of nobiletin concentrations (1.5–50 μM) exhibited an enhanced amplitude of PER2::LucSV and extended the rhythmic period in a dose- and *CLOCK*-dependent manner in mouse fibroblast cells [82]. In the same way, *BMAL1*-Luc human U-2 OS cells and *PER2*::LucSV mouse fibroblasts demonstrated rhythmic patterns in response to sudachitin (1–10 μM). Sudachitin notably enhanced the amplitude of *BMAL1* in a dose-dependent manner, ranging from 1.5- to 3-fold, and that of *PER2* at 3 and 10 μM, along with a period-lengthening effect of both *BMAL1* and *PER2* at the highest dose [85].

In a separate investigation on neuronal cells derived from *PER2*::Luc mice, it was documented that a low concentration of cinnamic acid (1 μM) sustained an amplitude for >5 d and shortened the period length of *PER2* in a dose-dependent manner (0.01–10 μM) [100]. In accordance with its potential implications on cardiac health, resveratrol (within the low concentration range of 0.25–2.5 μM) diminished the amplitude and lengthened the period of *PER2*-destabilized luciferase (dLuc) in a dose-dependent manner in neonatal rat cardiomyocytes (nrCMs). Unexpectedly, the data indicate that resveratrol can perturb the inherent rhythmicity of cardiac cells, potentially affecting their function [108]. Resveratrol, at a higher concentration of 50 μM, also advanced the phase and decreased the amplitude of *BMAL1* in mouse AML12 hepatocytes [101]. Curcumin administration at a concentration of 5 μM caused an average period of 24.48 h for *PER2* in rat C6 cells glioma cancer cells [88]. Apart from *PER2*, grape seed proanthocyanidin extract (100 mg/L) shifted the acrophase (the peak time of the rhythm) of virtually all core clock genes, including *CLOCK*, *BMAL1*, *CRY1*, *RORα*, and *Rev-Erbα*, in HepG2 cells. The extract also strengthened the amplitude of *Rev-Erbα* [91].

As for the potential influence of resveratrol on the rhythmic parameters of clock genes in relation to age changes, one study detected some alterations in synchronized human APCs obtained from young and old females. The treatment with a very high nonphysiological concentration (100 μM) of resveratrol led to an increase in the amplitude of *NR1D1* in both cells, whereas only *NR1D2* showing an amplified amplitude in young cells. It also caused a delayed phase of *NR1D1* and *NR1D2* in young and old cells, respectively, along with *PER1* in older cells. Despite *BMAL1*, *PER1*, *CRY2*, and *NR1D1/2* rhythmicity remaining unchanged in both cell types, resveratrol induced rhythmic oscillations of *CLOCK* and *CRY1*, accompanied by the loss of rhythmic patterns in *PER2*, *CRY2*, and *RORα*. It is noteworthy that its impacts diminished over the 48-h observation period, indicating a transient influence of resveratrol on the circadian rhythms of the preadipocytes [103]. Together, these studies demonstrate a role for (poly)phenols on the circadian rhythm characteristics of clock genes, including amplitude, period, and phase.

### Limitations and critical analysis of data

We have included findings regardless of the concentration used and the nature of the results, whether positive, negative, or conflicting. For example, 3 studies outlined in Table 2 [[67], [68], [69], [70], [71], [72], [73], [74], [75], [76], [77], [78], [79], [80], [81], [82], [83], [84], [85], [86], [87], [88], [89], [90], [91], [92], [93], [94], [95], [96], [97], [98], [99], [100], [101], [102], [103], [104], [105], [106], [107], [108], [109]], on cinnamic acid [100], naringin/naringenin [82], and catechins [106] reported no effects on the rhythmicity and/or expression of clock genes. One study showed that resveratrol disrupted the inherent rhythmicity of cardiac cells [108], whereas another study found no impact on the interaction between SIRT1 and CLOCK or BMAL1 [89]. Additionally, when exposed to 100 μM resveratrol, both young and old human APCs and human lung fibroblast cells exhibited varied responses. In old APCs, there were upregulatory effects on *BMAL1* mRNA [103], whereas in human lung fibroblast cells, *BMAL1* was increased in both young and old cells, with *PER1* showing elevated expression in old cells [95]. These data illustrate the complexity of drawing firm conclusions from the assessed studies. The observed variations in (poly)phenol implications could be attributed to several factors, such as the type and concentration of polyphenols used, cell type, and treatment conditions (i.e., transient or chronic).

The studies assessed here are methodologically heterogenous. Regardless of the presence or lack of cell synchronization, a number of studies elucidated the effects of (poly)phenols on cellular processes related to clock genes. It is important to note, however, that most studies utilized extremely high concentrations of (poly)phenols that are not physiologically relevant, even with supplementation. In general, (poly)phenols reach systemic circulation and target sites in the body at a concentration ≤5–10 μM [112,113]. Of the 16 studies considering their role in regulating cellular and metabolic processes, 14 used concentrations > 10 μM. Across all studies in this review, 35 studies deployed high concentrations of (poly)phenols above 10 μM.

Additionally, 12 studies have defined the timing of cell synchronization as zeitgeber time (ZT) instead of circadian time (CT), indicating a misunderstanding of term definitions. The term “zeitgeber time” refers to a measurement of time of an organism’s circadian rhythms based on zeitgebers such as light. However, “circadian time” is a term referring to a measurement of time of an organism’s endogenous circadian rhythms, independent of any external cues [114]. As aforementioned, in vitro cell models lack access to external cues compared with living organisms; accordingly, ZT is not applicable in this context.

Furthermore, sample collection time also varied across studies. According to the guidelines for circadian research, it is recommended that the collection of cell samples is initiated 24 h after the synchronizing agent has been removed to minimize the confounding effects of immediate early gene expression, which may be misinterpreted as part of the circadian cycle [63]. Thirteen of the 29 studies that used synchronized cells collected samples within 24 h of adding the synchronizing agent. Another issue is that 9 studies investigating the effect of (poly)phenols on clock gene and/or protein expression, irrespective of the use of cell synchronization, failed to mention the time points at which the reported changes were seen. This should be an essential requirement for any study, especially those exploring effects on circadian rhythms, because a key question lies in whether the observed changes are time dependent.

In terms of monitoring fluctuations of clock oscillators at transcriptional and translational concentrations, >50% of studies utilized chronological collection. This included sample collection for qPCR and Western blot, whereas a minority employed bioluminescence reporter technology. It is noteworthy that, as opposed to bioluminescence recording, the chronological sampling approach has certain limitations. Due to the absence of real-time detection of oscillations, temporal intervals between sample collections may result in the loss of critical peak or trough values. Furthermore, clock gene fluctuations at the single-cell level cannot be detected over time, which make it difficult to differentiate between intercellular synchronization or cell-autonomous clock function as the source of defects [115].

Many studies used diverse cancer and immortalized cell lines, many of which retain circadian rhythms. For example, HepG2 cells, widely employed in the publications reviewed here, exhibit less vigorous temporal variations in gene expression than primary cells but are valuable as in vitro models due to their ability to exhibit a rhythmic pattern of expression of clock genes in response to serum shock [116].

### Future prospects, recommendations, and take-home messages

**FIGURE 6:**
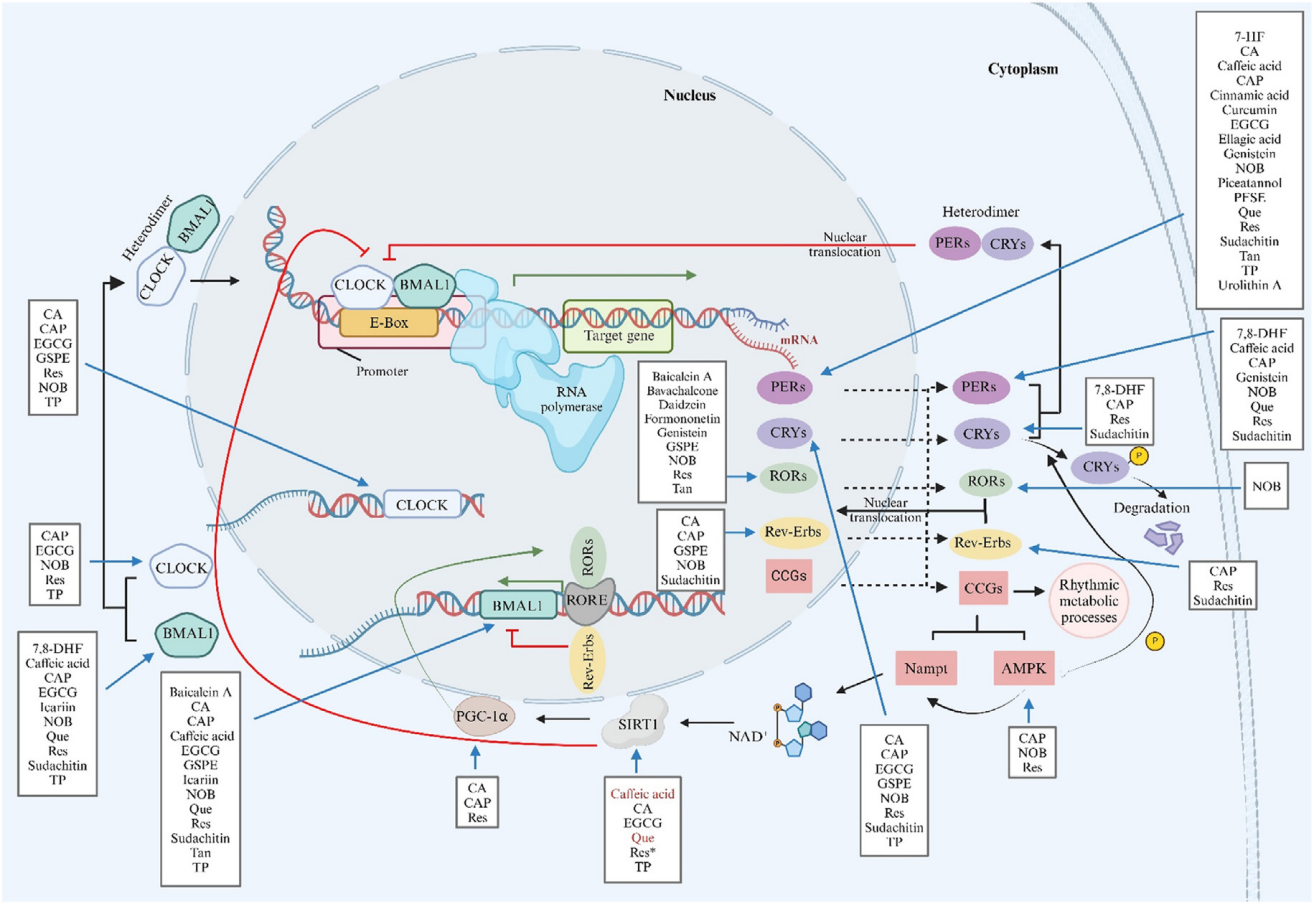
Putative sites of action for polyphenols targeting clock components. Information adapted from Huang et al. [15]. Abbreviations: AMPK, adenosine monophosphate–activated protein kinase; BMAL1, brain and muscle ARNT-like 1; CA, chicoric acid; CAP, capsaicin; CLOCK: circadian locomotor output cycles kaput; CCGs, clock-controlled genes; CRYs, cryptochromes; EGCG, (– )-epigallocatechin-3-gallate; GSPE, grape seed proanthocyanidin extract; NAD^+^, nicotinamide adenine dinucleotide; Nampt: nicotinamide phosphoribosyltransferase; NOB, nobiletin; P, phosphate group for phosphorylating and destabilizing CRY1; PERs: periods; PFSE passion fruit seed extract; PGC:1α, peroxisome proliferator–activated receptor gamma coactivator 1 alfa; Que, quercetin; Res, resveratrol; Rev-Erbs, reverse erythroblastosis viruses; ROR, retinoic acid–related orphan receptor; RORE, ROR enhancer elements; SIRT1: sirtuin1; Tan, tangeretin; TP, tea polyphenols. 7,8-DHF, 7,8-dihydroxyflavone. Blue arrow: the potential sites of action for polyphenols; dashed line: transcription–translation process; green arrow: upregulatory effect; red arrow: downregulatory effect; polyphenols in red color denote their effect on the SIRT1 transcript. Created with https://www.biorender.com/.

This is a complex and highly integrated area with multiple molecular pathways, combining metabolism, gene expression, and circadian processes. For that reason, we have included all data here from publications that were found in our systematic search on (poly)phenols and circadian processes in mammalian cells. We have commented on the concentrations used but not excluded any study because of this aspect. Figure 6 [15] depicts the known sites of action for (poly)phenols that target clock components to date from all of the studies.

In general, (poly)phenols are recommended to be tested below 5––10 μM concentration when carrying out in vitro experiments, because tissues will be exposed to this concentration as a maximum [112,113]. The exception is cells of the intestine, where the concentration in the lumen of the gut can be much higher. Another limitation of in vitro studies which should be pointed out is that phytonutrients rise and fall in blood concentration after consumption, and it is almost impossible to mimic this in vitro. Many studies, as can be seen from Table 2 [[67], [68], [69], [70], [71], [72], [73], [74], [75], [76], [77], [78], [79], [80], [81], [82], [83], [84], [85], [86], [87], [88], [89], [90], [91], [92], [93], [94], [95], [96], [97], [98], [99], [100], [101], [102], [103], [104], [105], [106], [107], [108], [109]], have used extremely high concentrations of (poly)phenols, even >200 μM. At this concentration, it is almost impossible not to see an effect, and the contribution of these studies to the field is questionable. In particular, resveratrol, although a highly bioactive compound, is only present in blood at 2.4 μM concentration even after a large dose (5 g) from a supplement [117]. Resveratrol is almost absent from most foods, and even in red wine, is only 0.2–5.8 mg/L [118]. In addition, most (poly)phenols are present in the blood as conjugates, with resveratrol sulfates and glucuronides as the main circulating species of resveratrol [119]. Tangeretin and nobiletin are notable exceptions, because they do not have hydroxyl groups available for conjugation. Although there are very few, if any, assessments of bioavailability of tangeretin and nobiletin in humans, nobiletin is well absorbed in rats owing to its hydrophobicity [[120], [121], [122], [123]], leading to concentrations in the blood in the low micromolar range. However, the concentration of nobiletin in the blood after consumption of citrus fruits or products is not known. Various (poly)phenols demonstrated the potential to improve rhythmicity of clock components at lower concentrations (≤10 μM) in multiple types of synchronized cells, including nobiletin, tangeretin, curcumin, bavachalcone, cinnamic acid, EGCG, resveratrol, and urolithin A. Notably, nobiletin at low concentration appears to function as a RORα/γ agonist, enhancing the rhythmic activity of *BMAL1* and exhibiting a positive amplitude–period correlation for *PER2*. Furthermore, it is evident across these studies that (poly)phenols target diverse clock components at mRNA and/or protein concentrations, in particular BMAL1, PER2, and RORα/γ. It is important to note that in future studies, it is preferable to synchronize cells, but the choice of synchronization approach depends on the cell type utilized and the advantages and disadvantages associated with various synchronizing agents. Samples should be collected at clearly defined time points 24 h after synchronization, to allow for noncircadian transient variations in gene expressions. Based on the publications assessed here, our recommendations for the future design of in vitro studies are listed in Table 3.

**TABLE 3:** Recommendations for future in vitro studies investigating the effects of (poly)phenols on circadian clock gene–mediated metabolic homeostasis in mammalian cells

1. Use an established synchronization method for all cell experiments.
2. Wait for ≥24 h after synchronization before collecting samples.
3. When treating with (poly)phenols, use physiologically relevant compounds and treat with doses ≤10 μM.
4. Measure gene expression and/or protein changes at multiple, clearly defined time points.
5. Use the correct nomenclature – circadian time (CT) – when reporting the timing of cell synchronization and treatments.
6. Treat with nobiletin as a positive internal control to help validate the experimental setup and ascertain that the observed rhythms are consistent with established reports.

Based on cell experiments, we conclude that future intervention studies on (poly)phenols and chronobiology should focus on nobiletin and/or tangeretin, initially on supplements until more information is available on bioavailability from foods and beverages containing citrus. Combined with a study on the timing of meals, it could be possible to ascertain if (poly)phenols could modulate to circadian processes in humans. Although meal timing and dietary macronutrients affect circadian processes, whether there is any additional effect from (poly)phenols in the food or potentially as supplements remains to be discovered.

## Author contributions

The authors’ responsibilities were as follows – GW, MJH, MPB: conceptualization; NS: methodology – initial search, writing – original draft preparation; NS, MJH, MPB, GW: data extraction; NS, MJH, GW: data charting; NS, MJH, MPB, GW: data curation; NS: visualization – production; GW: visualization – review and editing; GW, MJH, MPB: supervision; MJH, MPB, GW: writing – review and editing; and all the authors: contributed to the writing of the manuscript and approved the final version.

## Conflicts of interest

GW receives research funding from, and is a Scientific Advisor for, Nutrilite (Access Group, USA), and funding from the Product Makers, Australia, and the Australian Research Council. MPB receives funding from the National Health and Medical Research Council, Australia. The other authors report no conflict of interest.

## Funding

We thank King Abdulaziz University for a PhD Scholarship to NS.
