# Supplementary material for: Effects of (Poly)phenols on Circadian Clock Gene–Mediated Metabolic Homeostasis in Cultured Mammalian Cells: A Scoping Review
Source: Adv Nutr. 2024 Apr 20;15(6):100232. doi: 10.1016/j.advnut.2024.100232 (PMC11107464; doi:10.1016/j.advnut.2024.100232)
Supplement: Multimedia component 1 [file mmc1.docx]

**Supplementary Materials**

**Supplementary Table 1.** Full electronic search strategy for databases

| **Ovid Medline** | | |
| --- | --- | --- |
| **Concept** | **Key terms** | **MeSH subject headings** |
| **1** | (Polyphenol* OR Genistein OR Ellagi* OR apigenin OR Caffeic* OR curcumin OR daidzein OR Flavan* OR Flavon* OR Anthocyan* OR quercetin OR resveratrol).mp. OR *catechin*/ OR *cyanidin*/ OR Capsaicin.mp. OR Ferulic*.mp. OR Chlorogen*.mp. OR Hesperidin.mp. |  |
| **2** | (Circadian* OR Cry1 OR Cry2 OR Bmal1 OR CLOCK OR RORa OR Rev-erba OR rev-erb-alpha OR Per2 OR Per1 OR Periodicity OR ARNTL OR Melatonin OR Chronobiol* OR Chrononut* OR NR1D1 OR PERIOD2 OR Per3 OR (circadian* adj1 rhythm*) OR (Bmal1 adj1 gene*)).mp. | "ARNTL Transcription Factors"/ OR Circadian Clocks/ OR "circadian rhythm signaling peptides and proteins"/ OR exp arntl transcription factors/ OR exp clock OR proteins/ OR exp cryptochromes/ OR exp period circadian proteins/ OR  exp Periodicity/ OR exp Chronobiology Phenomena/ OR exp Cryptochromes/ |
| **3** | ((mammalian or human) adj2 cell line).mp. OR HepG2.mp. | exp Hepatocytes/ OR exp tumor cells, cultured/ OR exp cell line, tumor/ OR exp a549 cells/ OR exp caco-2 cells/ OR exp hep g2 cells/ OR Cell Line/ |

| **Scopus** | |
| --- | --- |
| **Concept** | **Key terms** |
| **1** | TITLE-ABS-KEY ( polyphenol* OR genistein OR ellagi* OR apigenin OR caffeic* OR curcumin OR daidzein OR flavan* OR flavon* OR anthocyan* OR quercetin OR resveratrol OR *catechin* OR *cyanidin* OR capsaicin OR ferulic* OR chlorogen* OR hesperidin ) |
| **2** | TITLE-ABS-KEY ( circadian* OR "Circadian rhythm" OR cry1 OR cry2 OR clock OR rora* OR {Rev-erba} OR rev-erb-alpha OR per2 OR per1 OR periodicity OR arntl OR melatonin OR chronobio* OR chrononut* OR nr1d1 OR period2 OR per3 OR ( bmal1 W/ 1 gene ) OR bmal1 ) |
| **3** | TITLE-ABS-KEY ( "Human cell line" OR "Mammalian cell line" OR hepg2 OR "Caco-2" OR hepatocyte* OR "Hepatoblastoma G2 cell line" OR "cell cultured" ) |

| **Web of Science Core Collection** | |
| --- | --- |
| **Concept** | **Key terms** |
| **1** | (((((((((((((((((((((ALL=(Polyphenol*)) OR ALL=(genistein)) OR ALL=(ellagi*)) OR ALL=(apigenin)) OR ALL=(caffeic*)) OR ALL=(curcumin)) OR ALL=(daidzein)) OR ALL=(flavan*)) OR ALL=(flavon*)) OR ALL=(anthocyan*)) OR ALL=(quercetin)) OR ALL=(resveratrol)) OR ALL=(capsaicin)) OR ALL=(ferulic*)) OR ALL=(chlorogen*))) OR ALL=(hesperidin))))) OR TS=(*cyanidin*)) OR TS=(*catechin*) |
| **2** | ((((((((((((((((((((((ALL=(circadian* )) OR ALL=( "Circadian rhythm")) OR ALL=(cry1)) OR ALL=(cry2)) OR ALL=(clock)) OR ALL=(rora*)) OR ALL=("Rev-erba")) OR ALL=("rev-erb-alpha")) OR ALL=(per2)) OR ALL=(per1)) OR ALL=( periodicity)) OR ALL=(arntl )) OR ALL=(melatonin)) OR ALL=(chronobio*)) OR ALL=(chrononut*)) OR ALL=(nr1d1)) OR ALL=(period2)) OR ALL=(per3))) OR ALL=( bmal1))) OR TS=( "bmal1 NEAR1 gene")) |
| **3** | ((((((((ALL=("Human cell line")) OR ALL=("Mammalian cell line")) OR ALL=(hepg2)) OR ALL=("Caco-2" )) OR ALL=(hepatocyte*)) OR ALL=( "Hepatoblastoma G2 cell line")) OR ALL=( "cell cultured")) OR ALL=("cell model")) OR ALL=("culture model") |
